# National and sub-national levels and causes of mortality among 5-19-year-olds in China in 2004-2019: A systematic analysis of evidence from the Disease Surveillance Points System

**DOI:** 10.7189/jogh.12.11008

**Published:** 2022-10-01

**Authors:** Yunning Liu, Yue Chu, Diana Yeung, Wei Wang, Lijun Wang, Peng Yin, Jiangmei Liu, Maigeng Zhou, Li Liu

**Affiliations:** 1 National Center for Chronic and Noncommunicable Disease Control and Prevention, Chinese Center for Disease Control and Prevention, Beijing, China; 2 Department of Sociology, The Ohio State University, Columbus, Ohio, USA; 3 Institute for Population Research, The Ohio State University, Columbus, Ohio, USA; 4 The Institute for International Programs, Department of International Health, Johns Hopkins Bloomberg School of Public Health, Baltimore, Maryland, USA; 5 Department of Population Family and Reproductive Health, Johns Hopkins Bloomberg School of Public Health, Baltimore, Maryland, USA

## Abstract

**Background:**

China accounts for 13% of the world’s 5-19-year-olds population. We estimated levels and trends of mortality by sex-age-cause among 5-19-year-olds at national and subnational levels in China annually from 2004 to 2019, to inform strategies for reducing child and adolescent mortality in China and other countries.

**Methods:**

We used adjusted empirical data on levels and causes of deaths from the China Center for Disease Control and Prevention’s Disease Surveillance Point (DSP) system. We considered underreporting and surveillance sampling design, applied smoothing techniques to produce reliable time trends, and fitted age-specific deaths and population to national estimates produced by international agencies to allow for cross-national comparisons.

**Results:**

The top leading causes for 54 594 deaths among 5-19-year-olds were neoplasms, road traffic injuries, and drowning. All-cause mortality in 5-19-year-olds has been declining steadily between 2004-2019, with evident yet narrowing geographical and gender disparities. Injury mortalities were one of the fastest declining causes, but widespread disparities were observed across subpopulations. Falling injuries and rising non-communicable diseases had the most pronounced epidemiological transition in the eastern region. Decrease in drowning fractions stalled for 15-19-year-olds in central/western rural areas. Suicide shares sustained or increased for 15-19-year-olds except among females in eastern rural areas.

**Conclusions:**

China made significant improvements in child and adolescent survival since 2004. However, constant targeted investments are needed to maintain and accelerate progress. A sustainable sample registration system like the DSP is likely essential for supporting such a process.

Older children (5-9-year-olds) and adolescents (10-19-year-olds) in China account for 13.3% of the world’s population in these age groups [1] · Since 1995, the government has prioritized reducing deaths from injury and infectious diseases through the “National Program for Child Development in China (2011-2020)” [2] and has also recently set goals for reducing premature mortality from non-communicable diseases through “Healthy China 2030 ”[3]. China has experienced steady improvement in child and adolescent survival, with one of the fastest mortality decrease among 5-19-year-olds in WHO West Pacific region [4,5]. To maintain and accelerate improvements in child and adolescent survival in the Sustainable Development Goals era [6], where well-being for all ages is emphasized, a close examination of how mortality for this age group has been declining in China could generate useful insights to inform other low- and middle-income countries (LMICs) [7].

A few existing studies provided evidence on all-cause and/or cause-specific mortality covering at least part of 5-19-year age group over the past decade in China [7-9]. Yet full time series of cause-specific estimates showing subnational patterns and disparities in changes haven’t been released by detailed cause-sex-age-breakdowns. To inform the health strategy in China and to link implications for reducing mortality in other LMICs, we provide in this paper the most up-to-date national and subnational levels and causes of mortality among 5-19 years in China annually between 2004 and 2019. The estimates are based on empirical data from the key disease surveillance system for all ages in China, the Disease Surveillance Points System (DSP).

## METHODS

### Design and sampling of the DSP system

The DSP System, housed in the China Center for Disease Control and Prevention (CDC), was the key sample registration system for disease surveillance and vital events for all ages in China. The surveillance points were selected using a multistage stratified clustered sampling design with stratification changing over time. DSP was initiated in the late 1970s with a few pilot sites, and first gained national coverage in 1990. In 2004, DSP was expanded to adapt to the rapidly changing mortality rate and gain representativeness at both national and subnational levels. Specifically, within each first-level stratum, namely Eastern, Central and Western regions, urban cities and rural counties were further stratified into 9-second-level strata by tertiles of economic status (defined by GDP for rural counties and level of urbanization for urban cities) and tertiles of population size. Existing sites were given priority to remain in the system, and new sites were purposively selected considering local capacity and geographic distribution. In total, 161 points (63 urban and 98 rural) were included in DSP, covering approximately 73 million (6%) population. Post-hoc evaluation studies confirmed representativeness at the national, regional, and urban/rural levels [10,11].

In 2013, the National Health and Family Planning Commission (previously known as Ministry of Health) combined the DSPs and vital registration (VR) system (hosted previously by Ministry of Health) to create an integrated national mortality surveillance system. Similar multistage stratified clustered sampling design was used. Within each of the 31 provinces as the new first-stage strata, all counties and cities were divided into eight second-stage strata based on urbanization, population size and crude death rate. Sample sizes were proportional to population size in the second-stage strata. Selection of sites were mostly purposive, largely keeping the original DSP sites while integrating 113 selected VR sites. After this expansion, DSP dramatically increased its coverage to nearly a quarter of the population (24.3% or 323.8 million) with a total of 605 surveillance points (208 urban and 397 rural points) and achieved provincial representativeness. More details of the system design are available in Appendix S1 in the **Online Supplementary Document** and previous publications [12-14].

### Cause of death collection

The identification and reporting of deaths and causes of deaths (COD) were conducted by DSP staff in district/county, city, and provincial level CDC [10,14]. Underlying COD on physician-filled death certificates were used for facility deaths. Deaths occurred outside health facilities were reported by village health workers to district/country CDC and a DSP staff would be dispatched to conduct household investigations and complete death certificates based on narrative medical history. No standardized verbal autopsy (VA) instruments were used until 2017 when WHO VA questionnaires were piloted in 13 selected DSP sites. COD were ascertained by physicians in township hospitals and coded using International Classification of Disease Codes (ICD-10). Data was cleaned, compiled, and reported monthly via computerized system by city, county, or provincial CDC. During the expansion and transition of DSP in 2004, data were retrospectively collected through the Third China National Cause of Death Sample Survey 2004-2005 [15]. Causes were grouped into IMPROVE categories (Appendix S2 in the **Online Supplementary Document**) adopted by WHO estimations [16] based on their ICD-10 codes, for analyses and data presentation.

### Quality control

Routine logic checking was conducted for quality control at the local and central CDCs. In addition, DSP has been conducting retrospective underreporting surveys every 3 years since 2009 in all sites, with the most recent survey done in 2018 reviewing data collected from 2015 to 2017. The design of the underreporting surveys has been evolving. (Appendix S3.1 in the **Online Supplementary Document**). In brief, all deaths occurring within 3 years prior to the survey year were captured through data triangulation by cross-referencing information from other government agencies such as Family Planning Offices and Civil Affairs Departments. Cases newly captured in underreporting surveys but not in original data were defined as missed deaths. They were followed up by household interviews for verbal autopsy.

We calculated under-reporting rates (URR) for each second-stage stratum annually for 2006-2017 as the proportion of missed deaths among the total number of deaths identified in under-reporting surveys for all 5+. (Appendix S3.1 in the **Online Supplementary Document**) We then derived under-reporting-adjusted all-cause and cause-specific estimates for all DSP sites by dividing DSP reported number of deaths by (1-URR). For 2018 and 2019, we used the latest available URR, ie, that for 2015-2017 to adjust for under-reporting. Data for 2004 and 2005 had quality control embedded in the retrospective survey data collection process [15], thus were not further adjusted.

### Statistical analyses

To generate nationally and sub-nationally representative death estimates for each sex and five-year age group, we accounted for the DSP sampling design after the underreporting adjustment. Sampling weights for each DSP site were derived from the total population under surveillance and the total population of the stratum which the surveillance site belongs to, assuming sites within each stratum having the same probability of being selected (Appendix S3.2 in the **Online Supplementary Document**) The under-reporting adjusted total number of deaths was weighted to get the all-cause and cause-specific number of deaths for each sex-age-group within each second-stage stratum.

For each second-stage stratum, the total number of deaths by age group, excluding natural disasters or collective violence, were first smoothed using linearly weighted 5-year moving average to reduce idiosyncratic noises in the time series. Then, adding back deaths due to natural disasters and collective violence, the totals were rescaled so that the national sum added up to the United Nations Interagency Group on Child Mortality (UN-IGME) crisis-free death envelope by age at the national level [5]. (Explanation of crisis and envelope is provided in Appendix S3.3 in the **Online Supplementary Document**) We subsequently applied five-year-moving-average-smoothed sex-split observed in DSP to IGME all cause deaths to derive sex-specific estimates for each stratum and age group. Similarly, age-sex-specific populations for second-stage stratum were also smoothed and rescaled to match the national totals produced by the United Nations Population Division for all years 2004-2019 [1].

Cause-specific numbers of deaths (CSND) for non-crisis COD were calculated for second-stage stratum for each age and sex by applying the cause-specific death fractions (CSDFs) to total non-crisis deaths derived above. The non-crisis CSDFs were smoothed using five-year-moving-average to reduce idiosyncratic noises in the time series, while fixing relative shares due to natural disaster and collective violence. Deaths due to 2008 Sichuan earthquake crisis were added to the crisis-free envelope for impacted provinces. (Details in Appendix S3.3 of the **Online Supplementary Document**) All-cause and cause-specific number of deaths and total population were then aggregated to the provincial, region-residency strata, and national levels step by step to produce national and subnational all-cause and cause-specific mortality estimates reported in this study. Rural and urban sites were defined by their naming convention (whether it was called an urban district or rural county) following the conventional DSP practice. A previous evaluation showed decent overall representativeness of the system for rural and urban areas [11]. Annual Rates of Reduction (ARR) were calculated to describe the average rate of decline in all-cause and cause-specific mortality over time (Appendix S3.4 in the **Online Supplementary Document**) [17].

The uncertainty estimates were generated using bootstrapping. Briefly, we re-sampled surveillance sites at random with replacement within each second-stage stratum for 2000 independent instances, from the pool of sites before and after the DSP system expansion separately. Uncertainty in all-cause death envelopes at the national level were also incorporated into the bootstrapping process by randomly drawing the death envelopes from distribution of IGME estimates. The 2 · 5th and 97 · 5th percentiles of the bootstrap distribution of possible observed causes-specific deaths estimates were considered as the lower and upper bounds for the final uncertainty range.

The analyses were performed using Stata/SE 15.1 [18], and data visualization were completed using ArcGIS10.6 [19] and R programming [20]. To promote transparency and replicability of global health estimates, the study complies with the Guidelines for Accurate and Transparent Health Estimates Reporting (GATHER) [21]. The GATHER checklist for this study can be found in Appendix S4 in the **Online Supplementary Document**.

## RESULTS

### National and sub-national all-cause mortality in 2019

**Figure 1:**
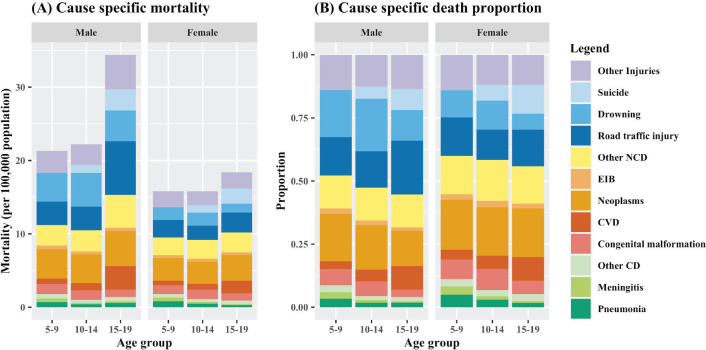
National cause-specific death fraction and mortality by age group, 2019. CVD – cardiovascular diseases, EIB – endocrine, immune, and blood diseases, CD – communicable, maternal, perinatal, and nutritional conditions, NCD – non-communicable disease. Small causes (with CSDF<0.025 in 2019) are not presented as individual causes in the graphs. More specifically, measles, TB, diarrhoea, and maternal causes are combined to “Other CD”; digestive causes are combined to “Other NCD”; legal violence, violence, war, and disaster are combined to “other injuries”.

In 2019, a total of 54 594 deaths occurred among 5-19-year-olds in China, with 16 167 (29.6%) occurring among 5-9-year-olds, 16 111 (29.5%) among 10-14-year-olds. and 22 316 (40.9%) among 15-19-year-lds. All-cause mortality rates were similar for 5-9-year-olds (18.7 per 100 000 population) and 10-14-year-olds (19.3 per 100 000 population), while significantly higher for 15-19-year-olds (27.0 deaths per 100 000 population). (**Figure 1**) For 5-14-year-olds, approximately 3 out of 5 deaths occurred among males. For adolescents, 7 out of 10 deaths were among males.

**Figure 2:**
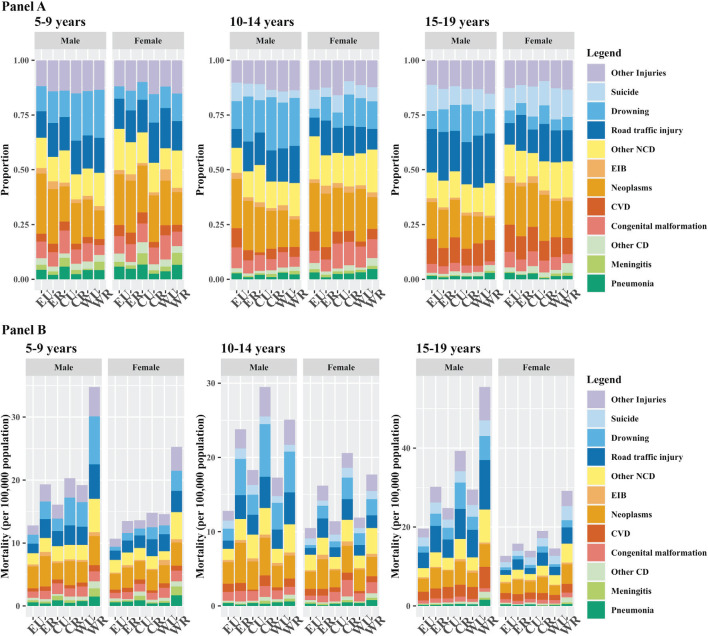
Strata cause-specific death fraction and mortality by age group, 2019. **Panel A**. Cause-specific death fractions. **Panel B**. Cause-specific mortality (per 100 000 population). EU – eastern urban, ER – eastern rural, CU – central urban, CR – central rural, WU – western urban, WR – western rural.

All-cause mortality showed apparent sub-national variations for all age-sex-groups (**Figure 2**). For 5-9-year-olds, all-cause mortality in the western rural stratum was significantly higher than that in other region-residency strata, with a 2 · 8 times difference compared to that in the eastern urban stratum for boys and 2 · 3 times difference for girls. Similar rural-urban disparities were observed for all 3 regions for 10-14, with the central rural stratum bearing slightly heavier mortality burden than the western rural stratum. For 15-19-year-olds, pronounced regional gradients were observed along with the urban-rural disparities. Western rural had over 84% higher mortality than eastern rural for both sexes, with all-cause mortality reaching 50 deaths per 100 000 population among males in western rural areas in 2019.

**Figure 3:**
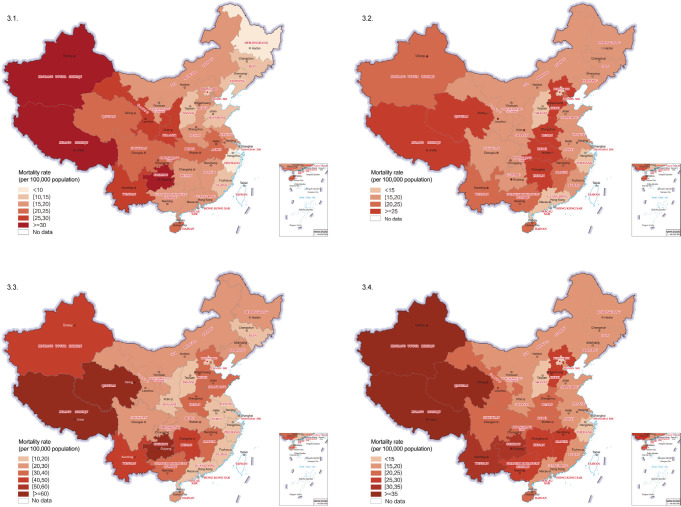
All-cause mortality by age and province in China in 2019. 3.1. 5-9-year-olds; 3.2. 10-14-year-olds; 3.3. 15-19-year-olds; 3.4. 5-19-year-olds.

Salient geographic difference in all-cause mortality was also found at the provincial level (**Figure 3**). Overall, the regional gradient in mortality burden was apparent, with the highest mortality provinces clustered in western and southwest regions of the country. The disparities in all-cause mortality between provinces were the greatest among 15-19-year-olds.

### National and sub-national causes of deaths in 2019

The leading causes were similar across age groups for 5-19-year-olds, with only small differences in relative shares (**Table 1**, **Figure 1**). Sex-specific differences in all-cause mortality were largely driven by non-suicide injury mortality. Neoplasm was the leading COD across 5–19-year-olds for females, consistently accounting for nearly one fifth of all deaths. For males, neoplasm was also the leading cause for 5-9-year-olds, while the relative importance gradually decreased among 10-19-year-olds. Injury-related causes, such as road traffic injuries, drowning, and other injuries, occupied 3 out of 5 leading causes for all age-gender groups which together contributed to around two fifths of total deaths. Drowning was more frequent among males and was the top COD for males aged 10-14 years. Road traffic injury gained predominance among males aged 15-19-year stacking up over one fifth of all deaths. Suicide, with persistent fractions in the past decade, replaced drowning as one of the top five COD for females aged 15-19-year-olds. The increase in relative shares of suicide was more noticeable for males aged 15-19 years. There was no obvious regional or rural/urban pattern in leading causes for these age groups, which implies that higher mortality in rural areas was driven by multiple causes rather than few particular causes (**Figure 2**, Table S3 in the **Online Supplementary Document**).

**Table 1:** National estimates of cause-specific death fractions, by sex and age group, 2019

5-9 y				10-14 y				15-19 y			
**Cause of death**	**CSDF**	**95% CI lower bound**	**95% CI upper bound**	**Cause of death**	**CSDF**	**95% CI lower bound**	**95% CI upper bound**	**Cause of death**	**CSDF**	**95% CI lower bound**	**95% CI upper bound**
**Male**											
Neoplasms	18.7	16.7	21.1	Drowning	20.8	19.1	22.4	Road traffic injury	21.3	20.0	22.6
Drowning	18.6	17.0	20.2	Neoplasms	17.7	15.9	19.7	Neoplasms	14.1	12.7	15.5
Road traffic injury	15.2	13.9	16.5	Road traffic injury	14.4	13.1	15.8	Other injuries	13.5	12.6	14.5
Other injuries	14.0	12.5	15.0	Ober NCD	13.1	11.9	14.3	Ober NCD	13.0	11.7	14.6
Other NCD	13.1	11.9	14.2	Other injuries	12.6	11.4	13.6	Drowning	12.1	10.9	13.3
Congenital malformation	6.4	5.6	7.4	Congenital malformation	5.9	5.0	6.9	CVD	9.3	8.5	10.1
Pneumonia	3.4	2.7	4.2	Suicide	4.8	4.1	5.7	Suicide	8.4	7.6	9.2
CVD	3.1	2.5	3.8	CVD	4.6	3.9	5.2	Congenital malformation	2.9	2.5	3.4
Other CD	2.8	2.3	3.3	Pneumonia	1.8	1.4	2.2	Pneumonia	1.8	1.2	2.5
Meningitis	2.5	2.0	3.1	EIB	1.8	1.4	2.2	Other CD	1.7	1.4	2.1
ElB	2.2	1.7	2.7	Other CD	1.6	1.2	2.0	EIB	1.3	1.0	1.6
**Female**											
Neoplasms	19.8	18.0	22.0	Neoplasms	19.1	17.0	21.2	Neoplasms	19.2	17.6	20.9
Road traffic injury	15.3	13.8	16.8	Other NCD	16.3	14.5	18.2	Other NCD	14.8	13.5	16.1
Other NCO	15.1	13.3	17.1	Road traffic injury	12.0	10.4	13.6	Road traffic injury	14.5	12.8	16.2
Other injuries	14.0	12.1	15.5	Other injuries	11.9	10.6	13.2	Other injuries	12.0	10.5	13.7
Drowning	10.7	9.2	12.2	Drowning	11.5	9.9	13.1	Suicide	11.4	9.9	13.0
Congenital malformation	7.8	6.8	8.9	Congenital malformation	8.4	7.1	10.0	CVD	9.4	8.3	10.6
Pneumonia	5.0	4.0	6.1	Suicide	6.2	5.1	7.4	Drowning	6.3	5.4	7.3
CVD	3.9	3.2	4.6	CVD	5.2	4.3	6.1	Congenital malformation	5.3	4.4	6.2
Meningitis	3.2	2.0	4.6	Pneumonia	3.0	2.1	4.0	Other CD	2.8	2.1	3.4
Other CD	2.9	2.3	3.6	EIB	2.5	1.8	3.3	EIB	2.0	1.5	2.4
EIB	2.2	1.6	2.9	Other CD	2.5	1.5	3.7	Pneumonia	1.7	1.3	2.2

CSDF – cause-specific death fractions, CI – confidence interval, CVD – cardiovascular diseases, EIB – endocrine, immune, and blood diseases, CD – communicable, maternal, perinatal, and nutritional conditions, NCD – non-communicable disease, y – years

### National and sub-national trend in all-cause mortality in 2004-2019

**Figure 4:**
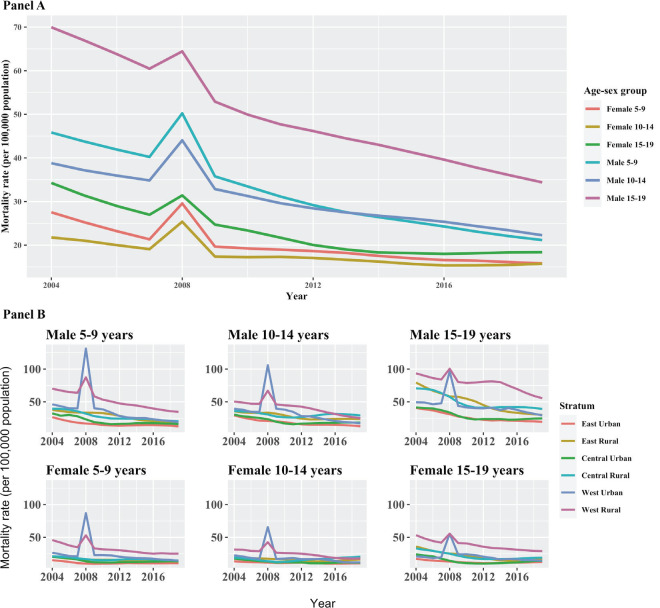
National and strata trend in all-cause mortality estimates, 2004-2019. The spikes in 2008 are due to natural disaster and crisis. (2008 Sichuan Earthquake). **Panel A**. National level. **Panel B**. Strata level.

**Figure 5:**
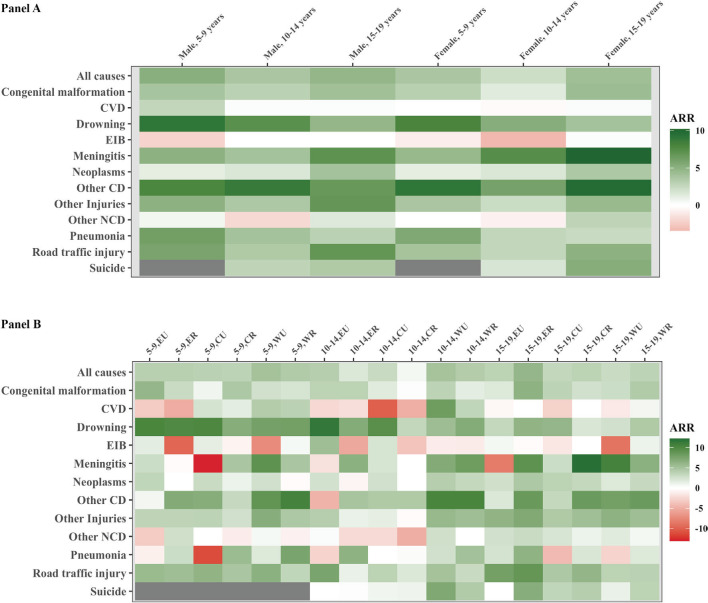
National and strata ARR heatmap on all-cause and cause-specific mortality, 2004-2019. **Panel A**. National level. **Panel B**. Strata level. Grey in cells indicates the all-cause or cause-specific mortality rate has a value of 0 in either 2004 or 2019. EA – eastern urban, ER – eastern rural, CU – central urban, CR – central rural, WU – western urban, WR – western rural.

All-cause mortality rates steadily decreased from 2004 to 2016 for all age-sex groups at the national level. (**Figure 4**, Panel A, **Figure 5**, Panel B; Table S4 in the **Online Supplementary Document**) Males experienced significantly higher yet faster-declining mortality than females for all age groups, leading to narrowing gender gaps across all years. The greatest and fastest decline was observed 5-9-year-old males, with an ARR of 5.1% per year, while the second greatest was observed between 15-19-year-olds, with a decrease in all-cause mortality by an ARR of 4.7% for females and 4.2% for males.

All-cause mortality generally decreased for most sub-national strata from 2004 to 2019 (**Figure 4****,** Panel B, **Figure 5****,** Panel B). Similar national gender pattern of higher ARRs for male were seen across strata and age groups. Decline in urban areas were mostly faster across regions for 5-14, opposite to 15-19-year-olds where rural declines were faster. (Table S4 in the **Online Supplementary Document**) The fastest declines were observed among 15-19-year-old males in the eastern rural stratum, with an ARR at 6.5%. The western rural stratum, though consistently having the highest or one of the highest mortality rates for all age-sex groups, has also been showing steady decrease across years. ARR for western rural ranged from 3.5% to 4.7% and the ARRs of western rural were higher than the national average for all age groups among females. The decrease in mortality rates of central rural and western urban areas slowed down in the past decade. Females aged 10-14 years old in the central rural area were also the only group with higher mortality in 2019 than 2004, increased from 19.3 to 20.5 per 100 000 population.

Great variations in both mortality levels and trends are found across and within provinces (Figure S1 and S2 in the **Online Supplementary Document**). Western provinces were consistently among the provinces with the highest mortality burdens. Provinces like Hubei experienced increased all-cause mortality in recent years. Progress toward improvement of adolescent survival also varies greatly across and within provinces. For some like Zhejiang or Shanxi, much progress has been made in reducing the gap between urban and rural areas; provinces like Sichuan and Guangdong had a decline in mortality in both urban and rural, areas yet the urban-rural gap remained. Ningxia, though overall showed remarkable decline in all-cause mortality, had its decline mostly concentrated in urban areas.

### National and sub-national trend in causes of deaths in 2004-2019

**Figure 6:**
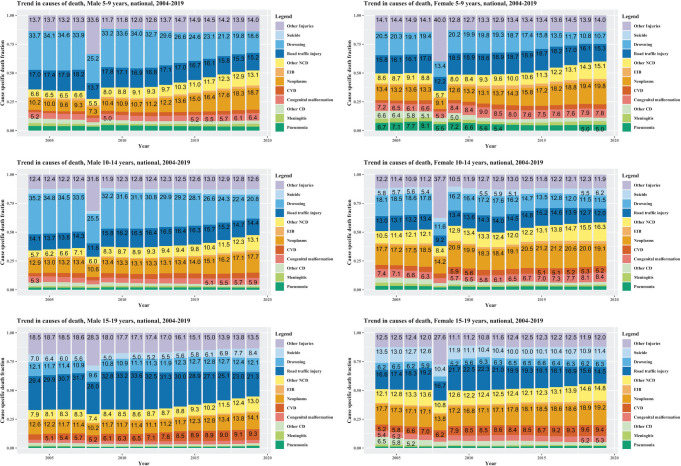
National trends of cause of death by age and gender, 2004-2019. Cells are labelled if CSDF are no less than 5.0%. The spikes in other injuries in 2008 are due to natural disaster and crisis (2008 Sichuan Earthquake).

The COD composition changed dramatically among 5-14 since 2004, yet it remained more stable among 15-19 over time (**Figure 6**). Males and females overall showed similar patterns in shifts in CSDFs. Non-communicable diseases, such as neoplasms, have gained increasing share since early- to mid- 2010s, and have gradually become the leading COD among all but 10-19 males. Shares of all injuries dropped dramatically for 5-14-year-olds, mostly attributable to the declines in drowning. Drowning was the leading cause for 5-14-year-old males, accounting for over one third of all deaths in 2004. Its CSDF dropped to around 20% in 2019. There were substantial declines in drowning among 5-14-year-old since 2010. For 15-19-year-olds, the contribution of road traffic injuries slightly increased till 2010 and had since been decreasing. While the ranking of suicide among females 15-19-year-olds dropped from the 3rd to 5th rank, while among males from 6th to 7th, its CSDF had hardly changed over time. In fact, the share of suicide showed steady increase since the early 2010s among 15-19-year-olds for both sexes.

The cause-specific mortality rates dropped for communicable diseases and injuries for all age-sex groups, with the highest ARR observed for drowning, meningitis, and other communicable diseases (**Figure 5**, Panel 2). Non-communicable diseases, such as cardiovascular, endocrine, immune, blood, and other non-communicable diseases, showed a much slower decline. Females 15-19 was the only group with mortality decreasing for all the COD categories.

### Sub-national trend in causes of deaths in 2004-2019

An epidemiological transition was observed in all region-residency strata during 2004-2019 (Figure S4 in the **Online Supplementary Document**). The transition in leading COD from injury-related causes to non-communicable diseases was most salient in the Eastern region, mostly driven by declining shares of injuries, namely drowning among older children, road traffic accidents among adolescents; as well as increasing shares of non-communicable diseases such as neoplasms and cardiovascular diseases. By contrast, the share attributable to drowning had stalled in the past decade among 15-19-year-olds in rural areas in the central and western regions. For females aged 15-19 years, a noticeable decrease was observed among other communicable diseases in western rural areas. The share of suicide sustained or increased among 15-19, with the only exception of decreasing observed among 15-19 females in eastern rural areas.

Regarding the CSMR, drowning and road traffic accidents were among the fastest declining CODs for most region-residence strata, with the ARR ranging from 2.1% to 11.5% for drowning and from 1.2% to 8.8% for road traffic injuries. In the east and central regions, urban areas usually saw faster decline in injury-related mortality than rural areas for 5-14-year-olds, while in the west region and for adolescents, the opposite was more likely (**Figure 5**, Table S4 in the **Online Supplementary Document**). Neoplasms, though with increasing shares in recent years, mostly showed low to moderate ARR in mortality over time. However, cardiovascular-specific mortality mostly decreased in the west region while increased in the other regions.

## DISCUSSION

China has made impressive progress in improving child and adolescent survival. Out of 195 countries in 2004-2019, its ARR in all-cause mortality for 5-9-year-olds, 10-14-year-olds, and 15-19-year-olds ranks 44, 54, and 23, respectively [5]. We speculate that the progress is partially driven by widespread economic development and its associated social advancement, especially in rural areas [7,22]. Backed by a strong economy, an unparalleled CNY82.1 billion were invested in adolescent health in China in 2014 alone [23]. Between 2004 and 2019, adolescent health and survival have been specifically outlined in major national development targets, such as the 2011 “China National Program for Child Development” and the 2016 “Healthy China 2030” planning outline [3,24], which ensures continued investment in and policy and program prioritization toward adolescent survival and health.

Continued and focused investments targeting a set of priority causes of deaths are key to further accelerate reduction in adolescent mortality. For example, neoplasms are an important cause of deaths among both males and females aged 5-19 years and their relative importance has been increasing from 2004 to 2019. The leading cancers in this age group include leukaemia and brain cancer, according to the International Agency for Research on Cancer estimates for 2018 for China (source: personal communications with Bochen Cao, technical lead at Divison of Data, Analytics and Delivery for Impact at WHO). Neoplasms have been declining at a moderate speed in 2004-2019. Since 2010, acute lymphoblastic leukaemia (ALL), which accounts for 80% of leukaemia in China, has been covered by the national insurance scheme. However, the insurance coverage for other cancers varies by geographies [25]. Cancer treatment could cause poverty. Expanding insurance coverage to major adolescent cancers could help afflicted families remain economically viable despite the socioeconomic and psychological burdens brought upon by the diseases.

Road traffic injuries are another major adolescent cause of deaths, the share of which has been declining in recent years especially among 15-19-year-olds. The pace of decline in road traffic injury specific mortality is at the medium level in 2004-2019. The decline could be partially attributable to improved transportation infrastructure [26]. Additionally, the national drunk driving law specifically requires the blood alcohol content limit among young and novice drivers to be below 0.02 g/dl with a high level of enforcement (9 out of 10 as rated by WHO) [27]. The success in reducing drunk driving-related road traffic injuries could serve as a model for multi-sectoral collaboration to address adolescent survival [28]. However, the enforcement of seatbelts was less successful and the seatbelt-wearing rate was only 37% according to a 2010 study among adults [27]. Clearly, enhanced enforcement of seatbelt wearing could be a low-hanging fruit among policy and program choices to improve adolescent survival, and continued collaborations and coordination between health and transportation sectors are required for effective execution.

Drowning is the third major adolescent cause of death, especially among 5-14-year-old males and particularly in rural compared to urban areas. Drowning-specific mortality rates have been declining at a fast pace especially among boys and girls aged 5-9 years and boys aged 10-14 years. Despite declining trends, drowning prevention has not been prioritized as much as road traffic injuries [29]. Interventions such as teaching school-aged children survival swimming skills and required use of personal flotation devices and provision of lifeguards at public swimming venues could be potentially effective in the Chinese context [30]. Interventions should be specifically targeted toward male rural adolescents and implemented in the Central and Western rural areas, where the decline in drowning-specific cause fractions has been stalled.

Comparing the COD distribution between boys and girls, one pronounced difference is in drowning, where boys had both higher drowning specific mortality rates and cause fractions than girls across all three 5-year age groups for the entire time-series estimated. It has been hypothesized that boys are at a higher risk of dying from drowning due to perceived better swimming abilities and more associated risk taking behaviours [29,31]. Boys also experienced higher mortality due to road traffic injuries and the gender difference was particularly stark among 15-19-year-olds when road traffic injuries specific mortality among boys was twice as high among girls in 2019, reflecting more riskier road use behaviours among boys than girls, and among those aged ≥15 years than those younger [32]. Suicide among 15-19-year-olds is relatively more important among girls than boys, which is consistent with studies on suicidal ideation [33]. Family conflicts, academic pressure, and teacher-student conflicts are reported as leading factors contributing to child and teenage suicide [34]. Interventions should clearly target boys to reduce mortality due to drowning and road traffic injuries, and 15-19-year-old girls due to suicide. School and family involvements should also be expected to play critical roles, especially in suicide prevention programs for adolescents.

In terms of differences across age groups, neoplasms, other non-communicable diseases, and other injuries are important across all three 5-year age groups. While the relative importance of road traffic injuries, suicide, and cardiovascular diseases increases with age, while that of drowning and congenital abnormalities decreases. Drowning prevention programs should clearly target younger age groups, whereas policies and programs related to road traffic injuries intervention, cancer, suicide, and non-communicable diseases should be focusing more on older adolescents and young adults.

Important and lasting geographic disparities exist in child and adolescent survival in China. The general pattern tracks the level of socio-economic development. In 2019, among 5-19-year-olds, the provinces with the highest all-cause mortality are all located in the socioeconomically least developed west and southwest regions, including Xinjiang, Qinghai, Xizang, Yunnan, and Guizhou. In contrast, majority of provinces in the east region, the most socioeconomically advantaged region, enjoy lower 5-19-year-olds all-cause mortality. However, there are some exceptions. For example, several central provinces, such as Hunan, Hubei, Henan, Hebei, had high levels of all-cause mortality among 10-14-year-olds, where Shaanxi and Gansu also experienced high levels of all-cause mortality among 5-9-year-olds. To comprehensively address under-development and improve survival health and well-being in Western China, the Chinese government adopted the Belt and Road Initiative in 2013 [35] and incorporated it into the constitution in 2017 [36] to prioritize economic and social development in Western provinces. The impact of this major initiative on child and adolescent survival is yet to be observed and evaluated.

Our estimates showed decent external validity comparing to estimates in other recent publications: Fadel et al. 2019 [8] based also on DSP data, and Dong et al., [9] derived from censuses data in China Statistical Yearbooks and Global Burden of Disease results. For 5-to-14-year-olds in 2016, our total deaths (34 721) were similar to Fadel’s estimates of 39 430, while 34% less than Dong et al. (52 861). Ours and Fadel’s had shared NCDs CSMF higher than Dong et al. by about 10%. The top 3 leading causes were the same across studies with similar CSDFs. Our estimates had slightly lower proportion for drowning (20.7% vs 24.3% for Dong et al. vs 22.1% for Fadel et al.) and higher proportion for neoplasms (17.1% vs 13.7% for Dong et al. vs 16.1% for Fadel et al.).

The study is subject to several limitations. First, 2004-2005 estimates were based on retrospective surveys, whereas 2006-2019 estimates were based on prospective surveillance. The two kinds of data collection mechanisms may cause incompatibilities in the time-series. However, no obvious bumps or dips were observed between 2005 and 2006 in our various time-series estimates. Second, site capacity was considered in the sample selection process. As a result, the post-hoc calculation for probability of being included in the sample may not fully capture the variance associated with the non-probability sampling, causing underestimation of uncertainty ranges. We attempted to address this partially by applying bootstrap technique to generate uncertainty. Third, due to low mortality in 5-19-year-olds, once we stratify DSP raw data by age, sex and geographic areas, some low-burden cause categories only had very few deaths. To address the small cell issue, we collapsed annual estimates into 4-year bins for the time-series. Fourth, provincial representativeness has only been achieved in the DSP after 2013. Therefore, the provincial time trends shown in the **Online Supplementary Document** should be interpreted with caution. Fifth, despite routine under-reporting in surveys since 2009, additional under-reporting is still likely. For example, in **Figure 4**, the western urban stratum has experienced some increase in age-sex-specific all-cause mortality, eg, around 2009. It is unclear whether such increases are real or due to improved reporting. We observed a similar pattern in our previous work on under-five mortality and speculated that the bump was due to improved data collection [37]. Lastly, the urban-rural classification was based on naming convention, which largely aligns with the actual urban-rural division. However, China has been experiencing rapid urbanization and the associated misclassifications could have caused underestimation of mortality in urban areas in certain provinces (eg, Xinjiang and Qinghai, see for example, Figure S5 in the **Online Supplementary Document**).

Nevertheless, the study also has several strengths. It is the latest study focusing on adolescents all cause and cause specific mortality using empirical data from China DSP. We provided detailed age-sex-cause-specific breakdowns for 5-19 at the national, regional, and subnational levels. The under-reporting and surveillance sampling design have been systematically considered and propagated in uncertainty estimation. We also fitted the level of all-cause age-specific mortality to the corresponding international estimates for the ease of global comparison.

Currently, information on all-cause and cause-specific adolescent mortality is extremely scarce in the LMICs, where most rely on periodic surveys for all-cause mortality and few had any empirical data on causes of deaths [5,16,38]. DSP sets a great example for other LMICs to consider setting up, maintaining, and strengthening similar sample registration systems to collect routine and timely cause of death data for the monitoring and evaluation of national and subnational adolescent survival policy and programs [39]. It is particularly relevant for countries in the process of developing such sample registration systems, eg, Mozambique [40] and Indonesia [41]. Key to the success of the DSP is sustained and renewed political commitment. For example, in 2014, three central government agencies, ie, the National Health and Family Planning Commission, Ministry of Public Security and Ministry of Civil Affairs, collectively issued an updated official document to further stress the significance of death registration. Meanwhile, data generated by the DSP have been extensively used to assess child and adolescent burden of diseases nationally and sub-nationally for research and policy purposes [29,42]. As China and other LMICs work to improve child and adolescent survival, a sustainable sample registration system, like the DSP, to support this process is essential.

## CONCLUSIONS

The study provides complete and most-up-to-date time series of mortalities by detailed geographic-age-sex- cause breakdowns for 5-19-year-olds in China, based on high-quality data from DSP. Close to 55 000 deaths occurred among 5-19-year-olds in China in 2019. All-cause mortality has been decreasing steadily for all age-sex groups. Higher ARR in mortalities for males and western rural areas lead to narrowing gender and geographical disparities over time. Cause of death distribution gradually shifted, with fast declining shares of injuries and increasing shares of non-communicable disease across country. Continued and targeted investments are needed to maintain and accelerate the progress. Sustainable sample registration system, like the DSP, also plays a critical role to support such process in LMICs.

## Additional material


Online Supplementary Document
