# Supplementary material for: National and sub-national levels and causes of mortality among 5-19-year-olds in China in 2004-2019: A systematic analysis of evidence from the Disease Surveillance Points System
Source: J Glob Health. 2022 Oct 1;12:11008. doi: 10.7189/jogh.12.11008 (PMC9526435; doi:10.7189/jogh.12.11008)
Supplement: Online Supplementary Document [file jogh-12-11008-s001.pdf]

National and subnational levels and causes of mortality among 5-19-year olds in China in 2004-2019 – a systematic analysis of evidence from the Disease Surveillance Points System

Yunning Liu[1*], Yue Chu[*2,3], Diana Yeung[4], Wei Wang[1], Lijun Wang[1], Peng Yin[1], Jiangmei Liu[1], Maigeng Zhou[1]‡, Li Liu[4,5‡]

[1] National Center for Chronic and Noncommunicable Disease Control and Prevention, Chinese Center for Disease Control and Prevention, Beijing, China
[2] Department of Sociology, The Ohio State University, Columbus, Ohio
[3] Institute for Population Research, The Ohio State University, Columbus, Ohio
[4] The Institute for International Programs, Department of International Health, Johns Hopkins Bloomberg School of Public Health, Baltimore, Maryland
[5] Department of Population Family and Reproductive Health, Johns Hopkins Bloomberg School of Public Health, Baltimore, Maryland

[*]indicates joint first-authorship
[‡]indicates joint corresponding and senior authorship

Appendix 1. DSP design, sampling, and data collection
Appendix 2. Additional details with analysis
Appendix 3. ICD-mapping of IMPROVE causes of deaths categories
Appendix 4. GATHER checklist
Appendix 5. Additional tables and figures (available in a separate file)

Appendix 1. DSP design, sampling, and data collection

The design and sampling of the DSP system evolved over time by roughly 4 stages: pre-1990, 1990-2003, 2004-2012, 2013-present.[1-4]

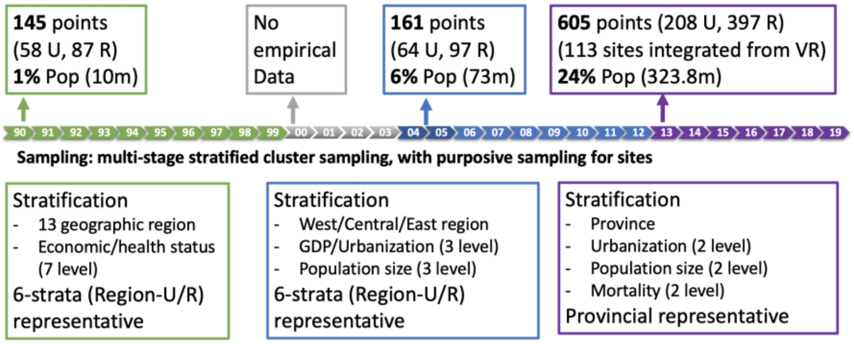

Abbreviation: Pop: population; U: urban; R: rural; m: million; VR: vital registration system.

The system first started with pilot sites of Dongcheng district and Tong county of Beijing in 1978. In 1989, 71 surveillance points in big cities and wealth rural areas from 29 out of 31 provinces volunteered to participate in disease surveillance work.[1]

In 1990, to gain national coverage and representativeness, the DSP system selected 145 surveillance points from all 31 provinces, using multi-stage stratified cluster sampling approach. All counties and cities were divided into 13 geographic regions, and then within each region, further stratified into 3 levels of urban and 4 levels of rural areas based on their economic and health status. Then urban communities and rural townships were selected as the surveillance points using probability proportionate to population size sampling (PPS) and systematic sampling in general, though with a few sites replaced considering the local capacity. A total of 58 urban points and 87 rural points were selected included in the DSP system, covering 10 million population. Since then, DSP has been generating estimates on levels and causes of mortality at national level and the six-region-residency-strata level (namely west rural, west urban, central rural, central urban, east rural, east urban). [2]

In 2004 and 2005, the DSP system were expanded again to adapt to the rapidly changing mortality rate and regain representativeness. The selection of new surveillance points followed a similar multi-stage stratified cluster sampling approach as the previous expansion. All rural counties and urban cities were divided into first-level strata based on region (east, central, west). Then within each first-level stratum, counties/cities were further stratified into 9 second-level strata by tertiles of economic status (GDP for rural counties and urbanization for urban cities) and tertiles of population size. Existing DSP sites were given priority to remain in the system, and new sites were purposively selected considering local capacity and ge[3]161 points (63 urban points and 98 rural points) were included in DSP covering 73 million population. Evaluation of the system showed decent general representativeness at national, regional, and urban/rural level as measured by a few indicators including crude birth and death rates and population composition. [3]

In 2013, the National Health and Family Planning Commission (NHFPC, previously known as Ministry of Health (MOH)) combined the DSPs and the vital registration (VR) system (hosted by previously MOH) to create an integrated national mortality surveillance system. [4] In this round of integration/expansion, disease surveillance points were selected using multi-stage stratified cluster sampling within each province. Within each of the 31 provinces in China (first-level strata), rural counties and urban districts were stratified into 8 second-level strata based on the following hierarchical structure:

1. high vs low urbanization, based on median urbanization index
2. high vs low population, based on median population size within each urbanization stratum
3. high vs low mortality, based on median total mortality rate within each urbanization-population stratum

The population indicators used in determination of the strata were from 2013 Statistical Yearbook of the Statistical Bureau of China. [7] A total of 248 province-residency-size-mortality strata were identified.

Then within each stratum, the number of surveillance sites needed for the aggregated system were sampled based on probability proportionate to population size. Conventional DSP sites were given priorities to be kept in the system, with 3 sites excluded due to low data quality and low local capacity to ensure data quality. Then 113 out of 319 sites from mortality surveillance sites in vital registration system were included. Factors like geographical balancing, not too advanced or lacking behind in human and financial resources, and local capability to manage and maintain long-term mortality surveillance work, were also taken into consideration in the final selection of surveillance sites. Along with 334 more newly selected surveillance points, current DSP dramatically increased its coverage to nearly a quarter of the population (24.3%, 323.8 million) with a total of 605 surveillance points (208 urban and 397 rural points), and achieved provincial representativeness for all estimates. [8]

Appendix 2. ICD-mapping of IMPROVE causes of death categories

Level	Category code	Cause name	ICD-10	ICD-9
1	AD1	Communicable, maternal, perinatal and nutritional conditions	A00-B99, D50-D53, D64.9, E00-E02, E40-E46, E50-E68, G00, G03-G04, H65-H66, J00-J22, N70-N73, O00-O99, P00-P96 (minus P23, P37.3, P37.4), U04	001-041, 045-139, 243, 260–269, 278, 279.5, 280-281, 285.9, 320-323, 381-382, 460-466, 480-487, 614-616, 630–676, 760-779, 783
2	AD2	HIV/AIDS	B20-B24	042-044,279.5
2	AD3	Diarrhea	A00, A03, A04, A06-A09	001, 004, 006-009
2	AD4	Measles	B05	055
2	AD5	Pertussis	A37	033
2	AD6	Typhoid	A01	002
2	AD7	Meningitis/encephalitis	A39, A83-A86, B94.1, G00, G03, G04	036, 049.0-.049.9, 062-064, 139.1, 320-323
2	AD8	Malaria	B50-B54	084
2	AD9	Lower Respiratory Infections	J09-J22, P23, U04	466, 480-487
2	AD10	Tuberculosis	A15-A19, B90	010-018, 137
2	AD11	Maternal causes	O00-O99	630–676
2	AD12	Other communicable, maternal, perinatal and nutritional conditions	Remainder of AD1	Remainder of Group AD1
1	AD13	Non-communicable diseases	C00-C97, D00-D48, D55-D89 (except D64.9), E03-E07, E10-E34, E65-E88, F01-F99, G06-G98 , H00-H61, H68-H93, I00-I99, J30-J98, K00-K92, L00-L98, M00-M99, N00-N64, N75-N98, Q00-Q99	140-242, 244-259, 270-279 (minus 279.5), 282-319 (minus 285.9), 324-380, 383-459, 470-478, 490-611, 617-629, 680-759
2	AD14	Congenital anomalies	Q00-Q99	740–759
2	AD15	Neoplasms	C00-D48	140–239
2	AD16	Cardiovascular disease	I00-I99	390-459
2	AD17	Endocrine, immune, and blood diseases	D55-D89 (except D64.9), E03-E07, E15-E34, E65-E88	240–242, 244-246, 251-259, 270-279 (except 274 and 279.5), 282-289 (except 285.9), 325, 415.1, 444, 451–453
2	AD18	Digestive (Gastrointestinal) disease	K20-K92	530-579
2	AD19	Other NCDs	Remainder of AD13	Remainder of AD13
1	AD20	Injuries	V01-Y09, Y35-Y36, Y40-Y86, Y88-Y89, Y87.0, Y87.1	E800-E978, E990-E999
2	AD21	Road traffic accidents	V01-V04, V06, V09-V80, V87, V89, V99	E800–E819, E826-E829, E929.0-E929.1
2	AD22	Drownings	W65-W74	E910
2	AD23	Natural disaster	X30-X39	E900-E909
2	AD24	Interpersonal violence	X85-Y09, Y87.1	E960-E969
2	AD25	Collective violence: legal intervention	Y35	E970-E978
2	AD26	Collective violence: war	Y36	E990–E999
2	AD27	Self-Harm	X60-X8, Y87.0	E950-E959
2	AD28	Other injuries	Remainder of AD20	Remainder of AD20
1	AD29*	Ill-defined	R00-R99, Y10-Y34, Y87.2	780-799, 980-989
2	AD30*	Injury intent not determined	Y10-Y34, Y87.2	980-989
2	AD31*	Other ill-defined	R00-R99	780-799

*Footnote: AD29 Ill-defined causes, and its subcategories AD30-AD31 are considered garbage codes in the study. Deaths with garbage codes were essentially proportionally redistributed to known causes excluding natural disaster and collective violence.

Appendix 3. Additional details with analysis

3.1 Underreporting adjustments

3.1.1 Underreporting survey

Apart from the double-checking and correction during the routine data reporting in local and central CDC, DSP have been conducting retrospective quality assessment and independent underreporting survey every 3 years since 2009 in all DSP sites, to review the completeness of reported deaths and causes of death for the 3 years prior to the survey. Townships and districts were selected following certain criteria, and all households in the selected areas were included in the household survey. All deaths occurred in 3 years prior to the survey year were identified and recorded by DSP staffs from multiple data sources: household interviews, local health facilities, Family Planning Offices, Maternal and Child Health Offices, Civil Affairs Departments, and Public Security Bureaus etc.. Cases not originally captured in routine DSP reporting were defined as missed cases, and causes of death and demographic information were collected for missed cases during the household interviews. Data were then electronically reported via the underreporting survey system and underlying causes were ICD-coded by local CDC.

3.1.2 Underreporting adjustment

The underreporting rates (URR) were calculated at provincial level annually for year 2006-2017 using the equation below.

$$\text{URR} = \frac{\text{\# missed cases newly identified in the underreporting survey}}{\text{\# all cases identified in the underreporting survey}}$$

No clear evidence showed that the data completeness and overall quality varied across causes, thus same URRs were applied to adjust all-cause and cause-specific number of deaths.

$$\text{\# URR adjusted deaths} = \frac{\text{\# case captured in routine DSP data}}{1 - \text{URR}}$$

3.2 Sampling probability calculation and data adjustment

We calculated the stratum-specific weights used to adjust the population and get subnational and national representative estimates for number of deaths by cause.

Table S1. Stratification for calculating sampling probabilities

Time period	1st-level strata	2nd-level strata
2004-2012	31 provinces	9-level based on GDP/urbanization and population size
2013-2019	31 provinces	8-level based on urbanization, population size and mortality level

Likelihood for being sampled for people from 2nd-level stratum j (Likelihood_j) for each age and gender group could be calculated using:

$$\text{Likelihood}_j = \frac{\sum_{i=1}^{m_j} Surveillance\ population_{i,j}}{Total\ population_j}$$

Where i denotes the ith surveillance point in stratum j, m_j is the total number of surveillance points in stratum j, $i \in (1,\ldots,m_j)$.

The total number of deaths within each stratum for each age and gender group could be calculated using:

$$\text{ATND}_j = \sum_{i=1}^{m_j} TND_i \times {}^{1}/_{Likelihood_j}$$

Where $ATND_j$ is adjusted total number of deaths for stratum j, TND_i is the total number of deaths for each surveillance point after adjusting for under-reporting.

3.3 Crisis estimates

3.3.1 Death envelope and crisis

All-cause death envelope is equivalent to all-cause number of death estimates, and is used interchangeably in this manuscript. It's usually used when fitting the cause-specific estimates "within" the all-cause estimates' boundaries, so the number from single causes would add up to match the all-cause totals. "Crisis-free" envelop would thus mean the total number of all causes except crisis.

Natural disasters and collective violence that severely impacted the population on a large scale are considered crisis in WHO-IGME estimates. The identification of crisis was case-by-case. 2008 Sichuan earthquake was identified as the only national natural disaster crisis during the period of 2004-2019 in China. Death estimates due to crisis are usually hard to capture, especially for countries relying on population-based surveys or sample registration system, where national estimates are inferred from sample of a small portion of the population. Therefore, crisis estimates directly from non-vital-registration data sources need to be used with cautious.

3.3.2 Subnational estimates for 2008 Sichuan earthquake

Given that DSP wasn't designed to fully cover all urban and rural areas that was affected by earthquake, and considering the challenges in cause of death data collection and verbal autopsy survey post-disaster via surveillance system, we derived natural disaster estimates for four provinces affected by this crisis – Sichuan, Shaanxi, Gansu and Chongqing, from IGME age-specific crisis estimates, for year 2008.

IGME total deaths exceeds the non-crisis deaths envelope were identified as national age-specific crisis death for year 2008. Then these deaths were redistributed to Sichuan, Shaanxi, Gansu and Chongqing provinces proportional to total deaths reported in latest available government reports and mainstream news in these provinces. [5-9] Yunnan, Henan, Hubei, Guizhou and Hunan had reported deaths due to earthquakes, but was either not in age groups of interest, [10-12] or was thus were not included in the crisis estimation.
Then for urban and rural split of the deaths, we imputed percentage of urban earthquake deaths from sub-regional reporting when available, [6] otherwise used percentage of urban population instead. For Sichuan, we treated earthquake deaths in cities as urban deaths. [5] For Shaanxi, deaths in cities with top 5 GDP per capita in province as urban deaths. [6] For Chongqing, we calculated the proportion based on deaths with location information available. [8] For Gansu, we used percentage of urban population for 2008. [13] Then we used the 5-year-moving-average smoothed gender splits to further breakdown the estimates by gender.

Table S2. Subnational breakdowns for excess deaths due to 2008 Sichuan Earthquake

Province	Total earthquake death	Percent in national crisis death	Percent deaths in urban area
Sichuan	68660	99.27%	45.56%
Shaanxi	122	0.18%	48.36%
Gansu	364	0.53%	60.95%
Chongqing	16	0.02%	45.45%

3.4 Annual Rate of Reduction (ARR)

The annual rate of reduction (ARR) was calculated by:

$$\mathrm{ARR} = \frac{\ln\left(\frac{\mathrm{MR}_{last\ year}}{MR_{first\ year}}\right)}{\text{Last year} - \text{First year}} \times 100 \times (-1)$$

Appendix 4. GATHER checklist

Item	Checklist item*	Section(s) or sources providing information
**Objectives and funding**		
1	Estimated indicator and population	Methods
2	Funding sources	Funding section of abstract
Data Inputs		
For all data inputs from multiple sources that are synthesized as part of the study:		
3	Data identification	Methods and appendix 1
4	Inclusion/exclusion criteria	Not applicable
5	Included data sources and their main characteristics	Methods and appendix 1
6	Potential important biases of input data	Discussion and appendix 1,2
For data inputs that contribute to the analysis but were not synthesized as part of the study:		
7	Source of other data inputs	Methods
For all data inputs:		
8	Accessible input data files	Open access databases**
Data analysis		
9	Conceptual overview of the data analysis method	Methods and open access databases**
10	Description of all steps of the analysis	Methods and appendix 1-3
11	Model selection methods	Not applicable
12	Model performance and/or sensitivity analysis.	Not applicable
13	Uncertainty estimation methods	Methods and discussion
14	Statistical code	Open access databases**
Results and discussion		
15	Accessible estimates data files	Open access databases**
16	Uncertainty of the estimates	Results and discussion
17	Results interpretation in light of existing evidence	Results and discussion
18	Limitations of the estimates	Discussion

*Detailed GATHER statement with explanation and elaboration of the items can be found on gather-statement.org

** Open access databases with input files and analytical code of the study can be accessed on github https://github.com/y-chu/China_5-19COD

Reference

1. Yang G. Selection of DSP points in second stage and their presentation. Chinese Journal of Epidemiology [Zhonghua liuxingbingxue zazhi]. 1992;13(4):197.
2. Yang G, Hu J, Rao KQ, Ma J, Rao C, Lopez AD. Mortality registration and surveillance in China: history, current situation and challenges. Popul Health Metr. BioMed Central; 2005;3(1):1–9.
3. Zhou M, Jiang Y, Huang Z, Wu F. Adjustment and representativeness evaluation of national disease surveillance points system. Disease Surveillance. Editorial Board of Disease Surveillance; 2010;25(3):239–44.
4. Liu S, Wu X, Lopez AD, Wang L, Cai Y, Page A, et al. An integrated national mortality surveillance system for death registration and mortality surveillance, China. Bull World Health Organ. 2016;94(1):46–57.
5. Tencent news. Summary of Sichuan earthquake deaths [Internet]. 2008 [cited 2021 Apr 16]. Available from: https://news.qq.com/zt/2008/dizhen/newdata.htm
6. CCTV. 122 people died and 24,300 houses collapsed in rural areas in Shaanxi due to Sichuan earthquake [Internet]. 2008. Available from: http://news.cctv.com/china/20080613/108871.shtml. Accessed: Apr 16 2021
7. CCTV. Summary of Sichuan earthquake deaths. 2008. Available from: http://news.cctv.com/china/20080513/100052.shtml. Accessed: Apr 16 2021
8. The State Council Information Office of the Peoples Republic of China. Press Conference for earthquake damage of Chongqing [Internet]. 2008. Available from: http://www.scio.gov.cn/xwfbh/gssxwfbh/xwfbh/chongqing/document/317451/317451.htm. Accessed: Apr 16 2021
9. Phoenix Television. Summary of Sichuan earthquake deaths [Internet]. 2008. Available from: http://news.ifeng.com/special/0512earthquake/ Accessed: Apr 16 2021
10. Xinhua News Agency. Wenchuan Earthquake affected Yunnan Zhaotong, 1 dead and 9 injured [Internet]. 2008 [cited 2021 Apr 16]. Available from: https://news.ifeng.com/c/7fYp4h3wTwU
11. China News Service. Sichuan earthquake in Henan, 2 died and 5 injured [Internet]. 2008. Available from: http://news.sina.com.cn/c/2008-05-13/130215529343.shtml Accessed: Apr 16 2021
12. Xiaoxiang Morning News. People who died in Changde earthquake received 80,000 yuan in funeral expenses [Internet]. 2008. Available from: http://epaper.xxcb.cn/xxcba/html/2008-05/20/content_33810.htm Accessed: Apr 16 2021
13. Bureau of Statistics. Demographic characteristics of Gansu in 2008 [Internet]. 2009. Available from: http://www.stats.gov.cn/ztjc/ztfx/dfxx/200903/t20090323_34708.html Accessed: Apr 16 2021

National and subnational levels and causes of mortality among 5-19-year olds in China in 2004-2019 – a systematic analysis of evidence from the Disease Surveillance Points System

Appendix 5. Additional Tables and Figures (continued)

- Figure S1. Provincial trend in all-cause mortality estimates in 2004-2019
- Figure S2. ARR for all cause mortality by province
- Figure S3. Provincial level ARR heatmaps
- Figure S4. Strata trends of cause of deaths (COD) by age and gender in 2004-2019
- Figure S5. Province trends of cause of deaths (COD) in 2004-2019
- Table S3. Region-residency strata level cause-specific death, fraction and mortality estimates, 2019
- Table S4. National and subnational annual rate of reduction (ARR) by age and sex
    - Table S4.1. National all cause and cause-specific ARR
    - Table S4.2. Regional-residency strata level all cause and cause-specific ARR

This documents presents additional figures and tables.

Figure S1. Provincial trend in all-cause mortality estimates in 2004-2019

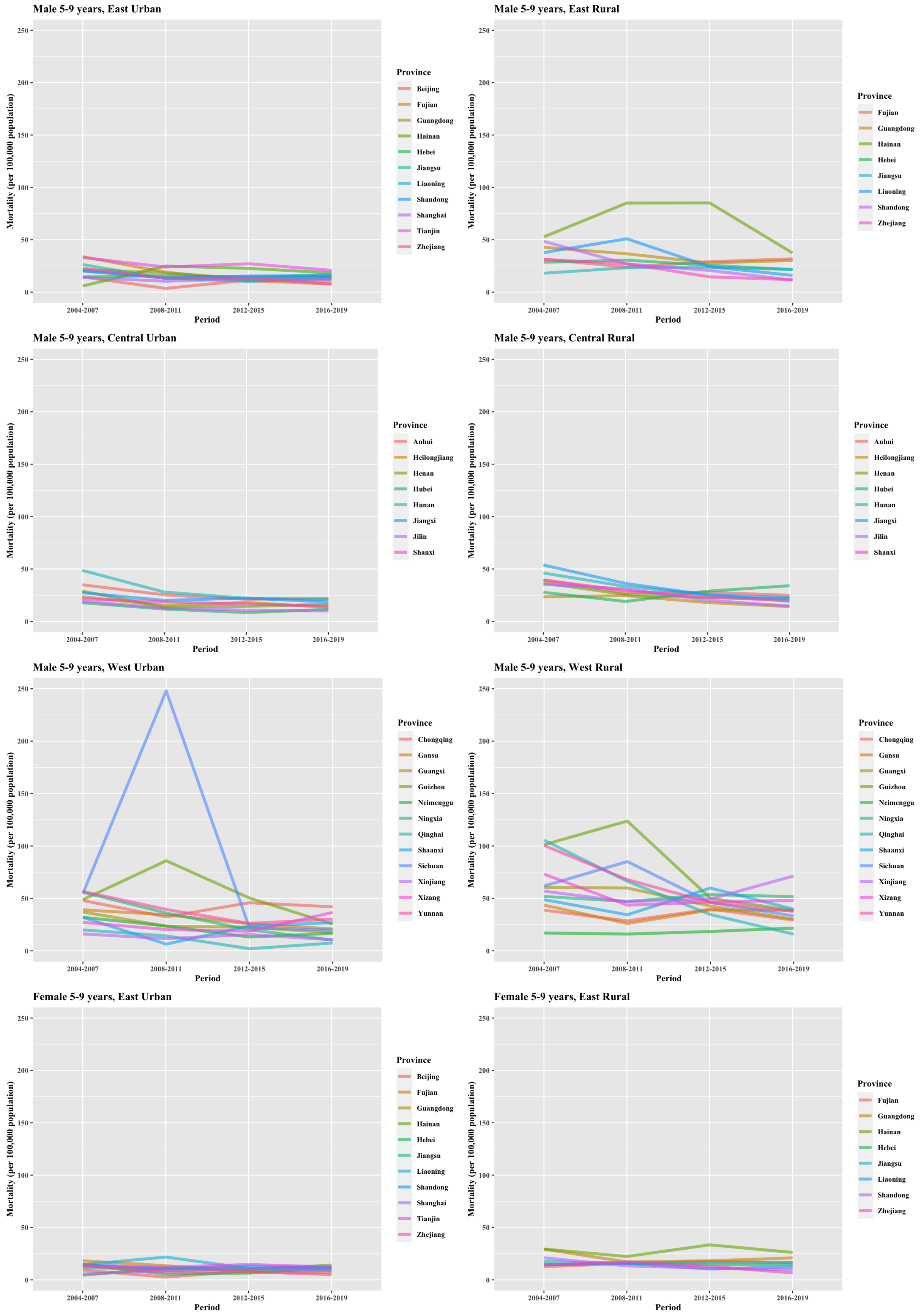

Female 5-9 years, Central Urban

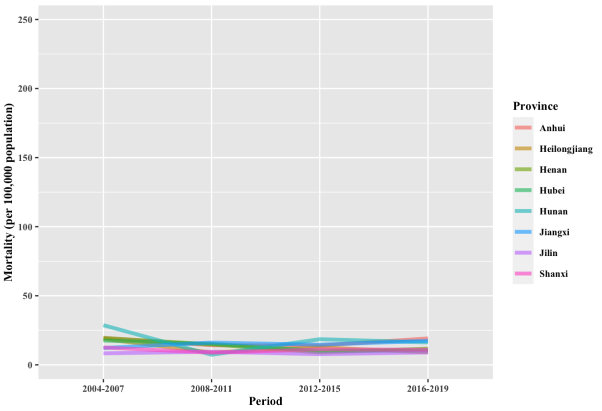

Female 5-9 years, Central Rural

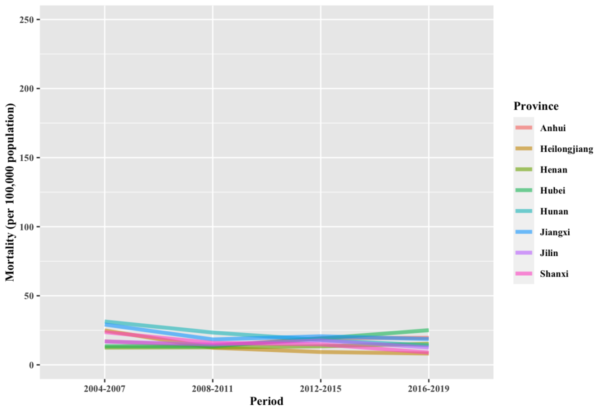

Female 5-9 years, West Urban

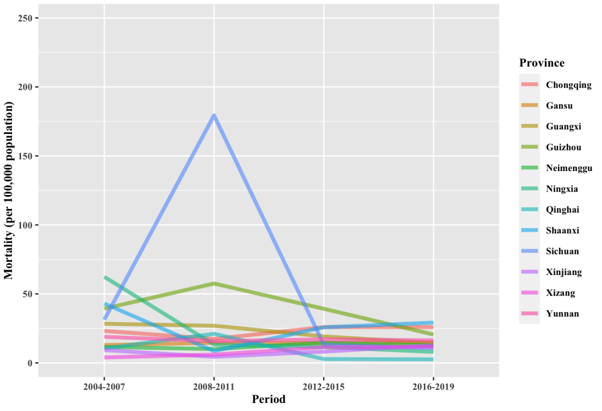

Female 5-9 years, West Rural

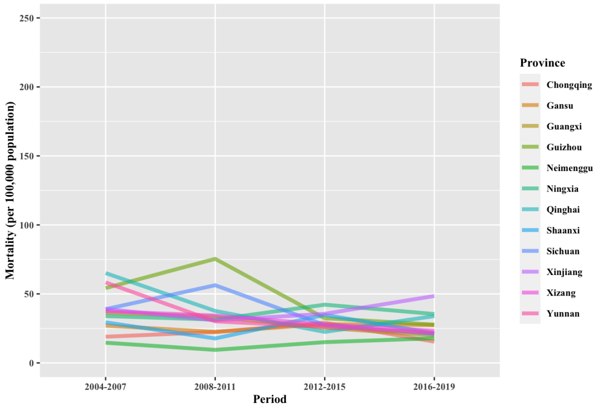

Male 10-14 years, East Urban

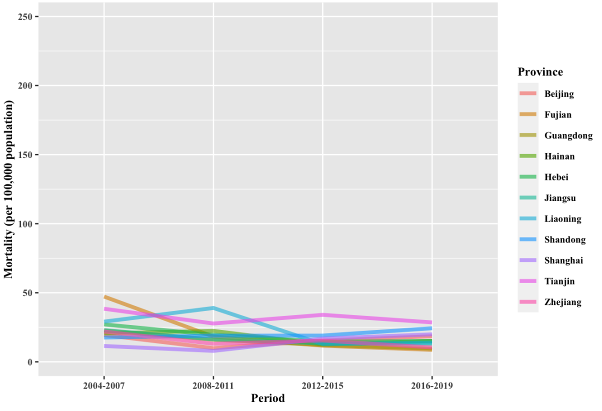

Male 10-14 years, East Rural

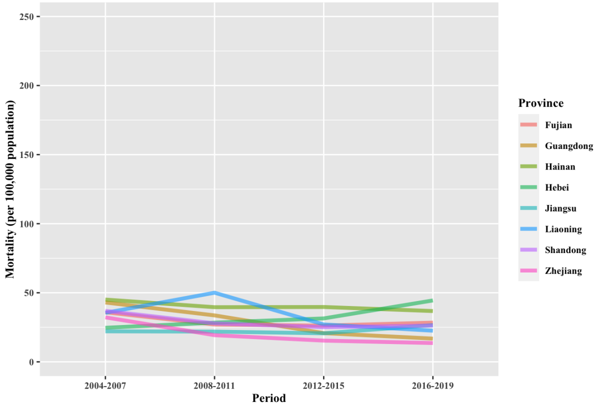

Male 10-14 years, Central Urban

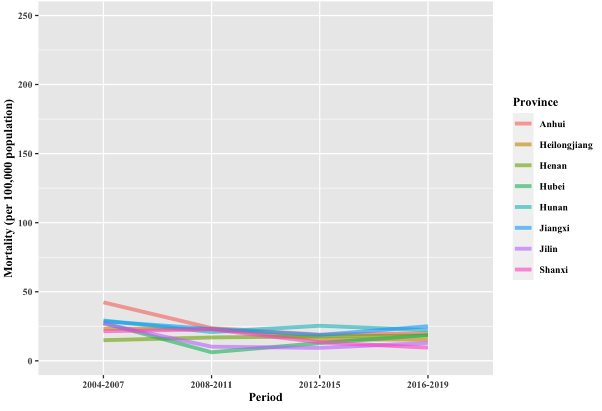

Male 10-14 years, Central Rural

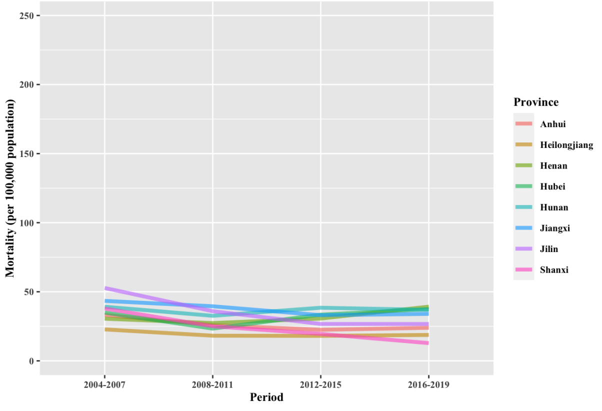

Male 10-14 years, West Urban

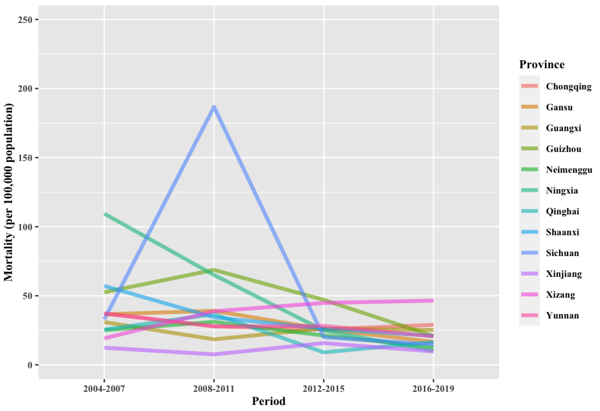

Male 10-14 years, West Rural

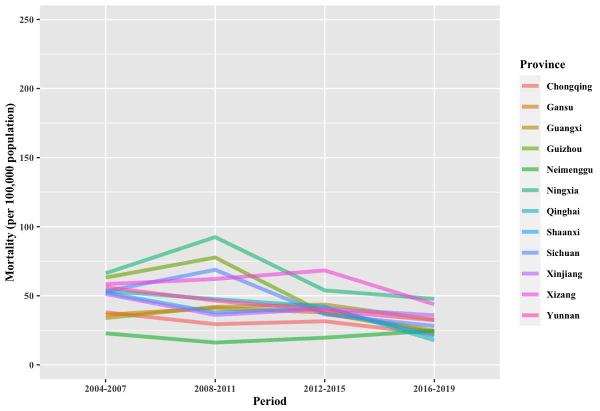

Female 10-14 years, East Urban

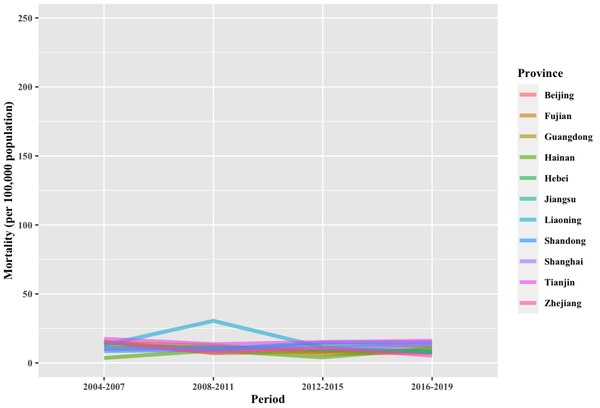

Female 10-14 years, East Rural

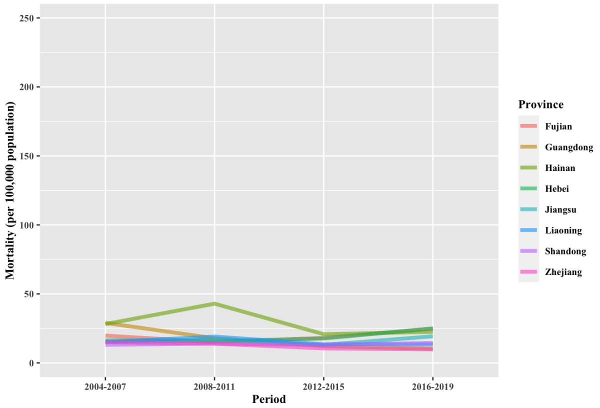

Female 10-14 years, Central Urban

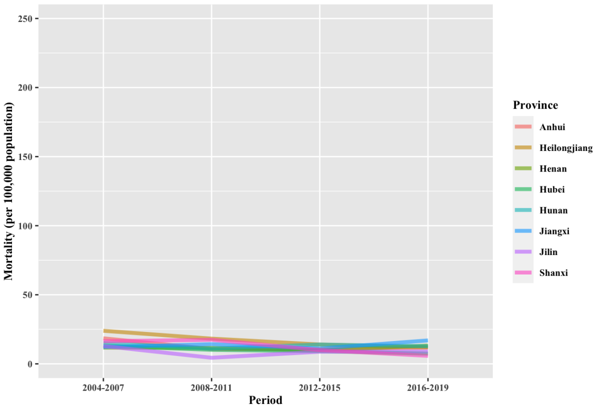

Female 10-14 years, Central Rural

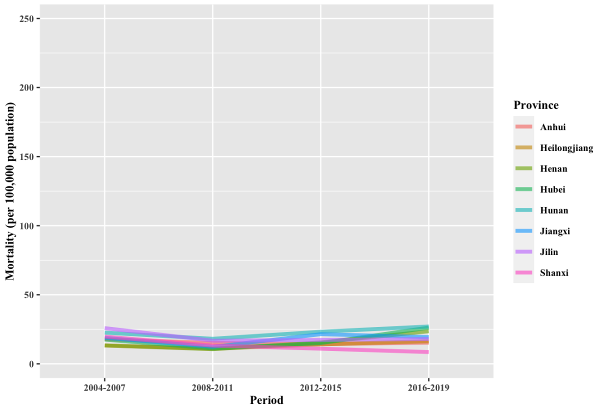

Female 10-14 years, West Urban

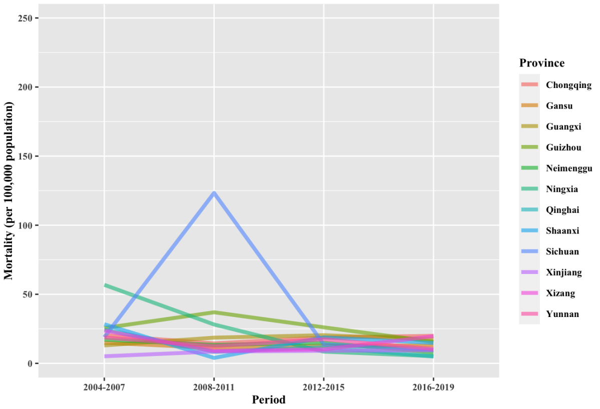

Female 10-14 years, West Rural

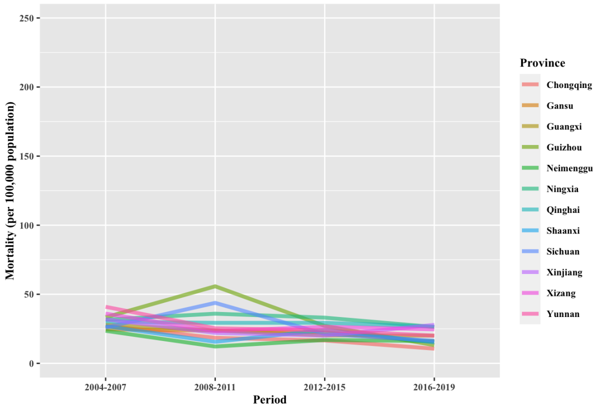

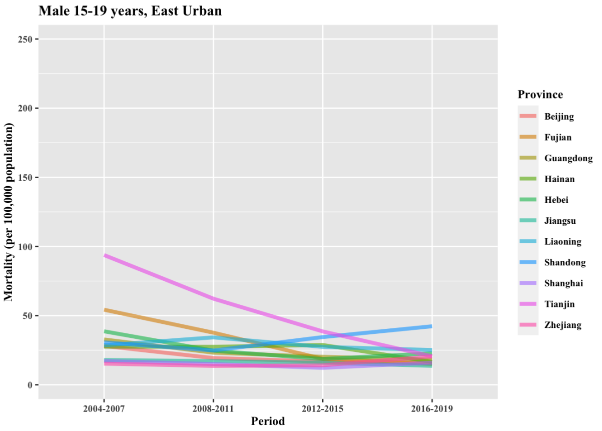
Male 15-19 years, East Urban
Mortality (per 100,000 population)
Period
2004-2007
2008-2011
2012-2015
2016-2019
Province
Beijing
Fujian
Guangdong
Hainan
Hebei
Jiangsu
Liaoning
Shandong
Shanghai
Tianjin
Zhejiang

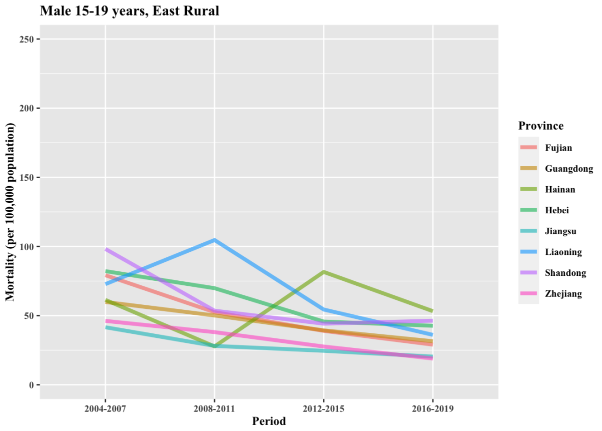
Male 15-19 years, East Rural
Mortality (per 100,000 population)
Period
2004-2007
2008-2011
2012-2015
2016-2019
Province
Fujian
Guangdong
Hainan
Hebei
Jiangsu
Liaoning
Shandong
Zhejiang

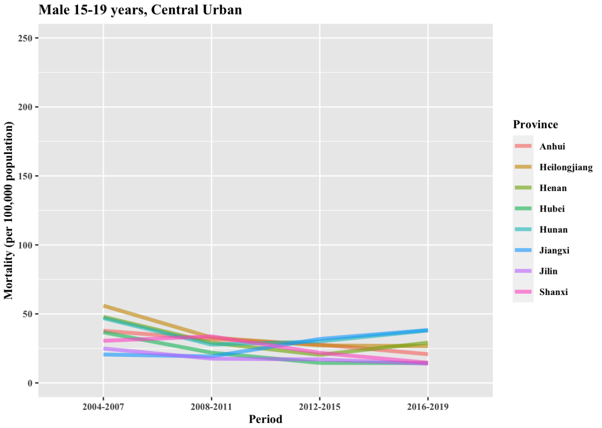
Male 15-19 years, Central Urban
Mortality (per 100,000 population)
Period
2004-2007
2008-2011
2012-2015
2016-2019
Province
Anhui
Heilongjiang
Henan
Hubei
Hunan
Jiangxi
Jilin
Shanxi

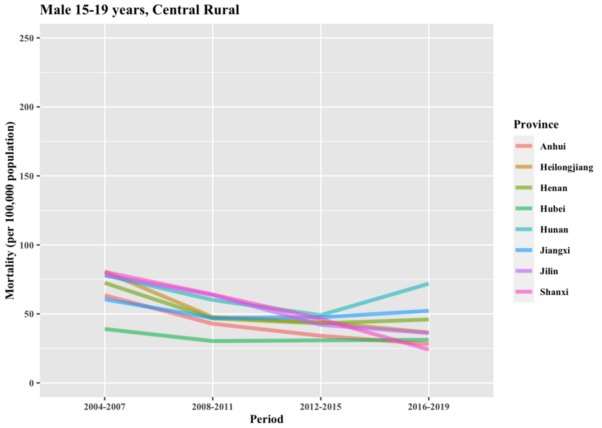
Male 15-19 years, Central Rural
Mortality (per 100,000 population)
Period
2004-2007
2008-2011
2012-2015
2016-2019
Province
Anhui
Heilongjiang
Henan
Hubei
Hunan
Jiangxi
Jilin
Shanxi

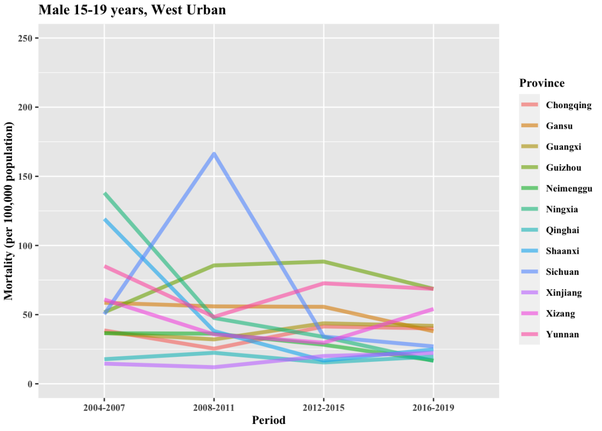
Male 15-19 years, West Urban
Mortality (per 100,000 population)
Period
2004-2007
2008-2011
2012-2015
2016-2019
Province
Chongqing
Gansu
Guangxi
Guizhou
Neimenggu
Ningxia
Qinghai
Shaanxi
Sichuan
Xinjiang
Xizang
Yunnan

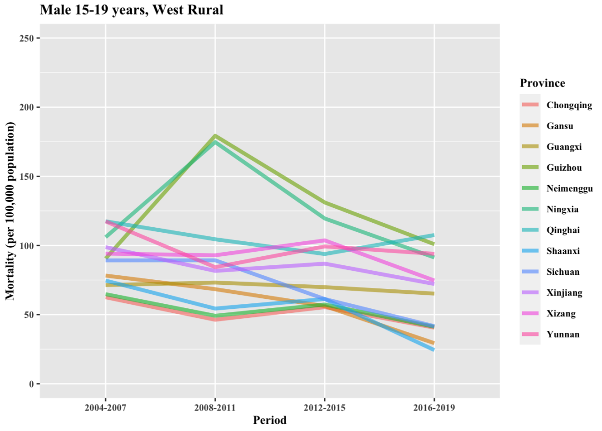
Male 15-19 years, West Rural
Mortality (per 100,000 population)
Period
2004-2007
2008-2011
2012-2015
2016-2019
Province
Chongqing
Gansu
Guangxi
Guizhou
Neimenggu
Ningxia
Qinghai
Shaanxi
Sichuan
Xinjiang
Xizang
Yunnan

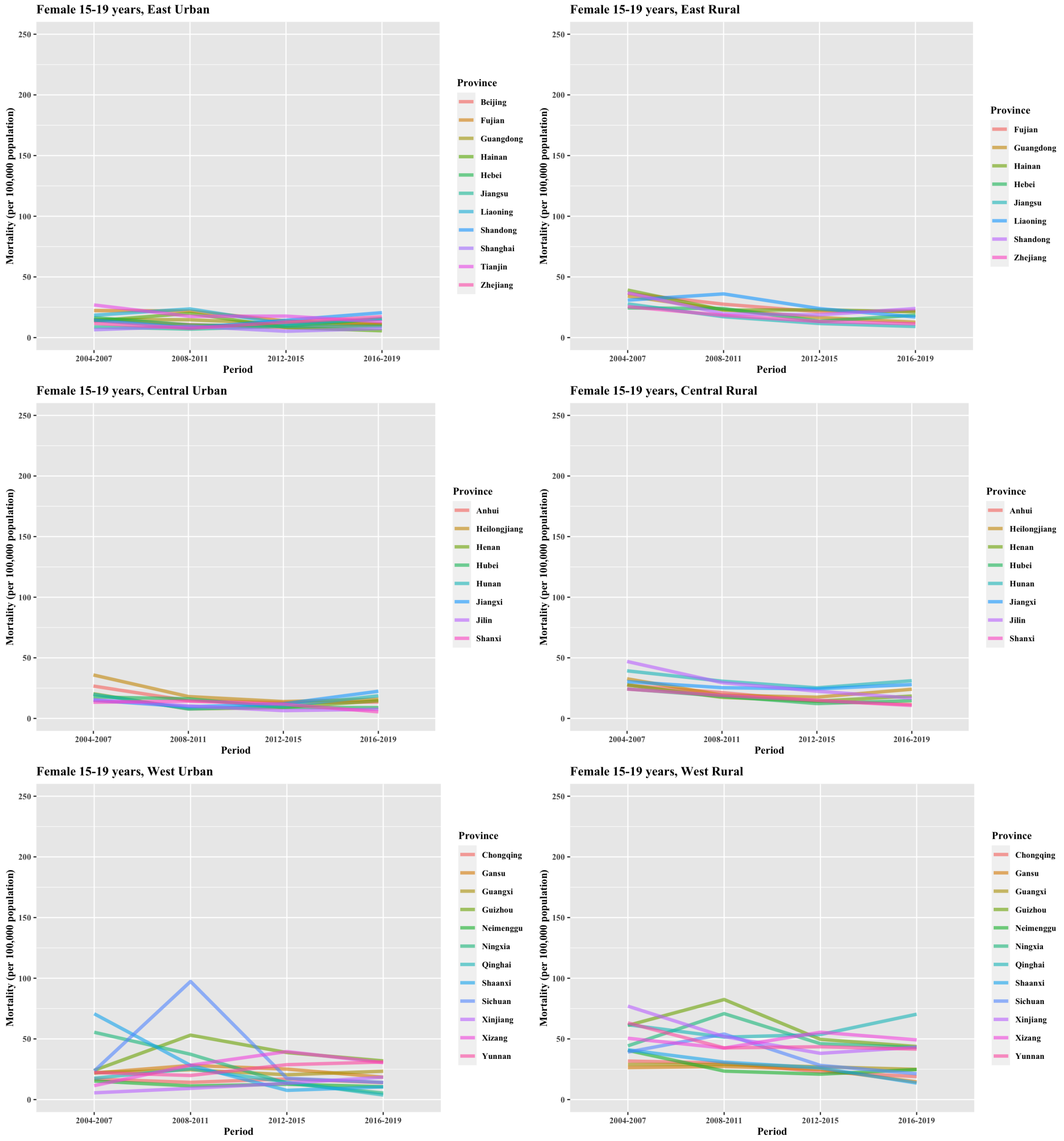

Figure S2. ARR for all cause mortality by province

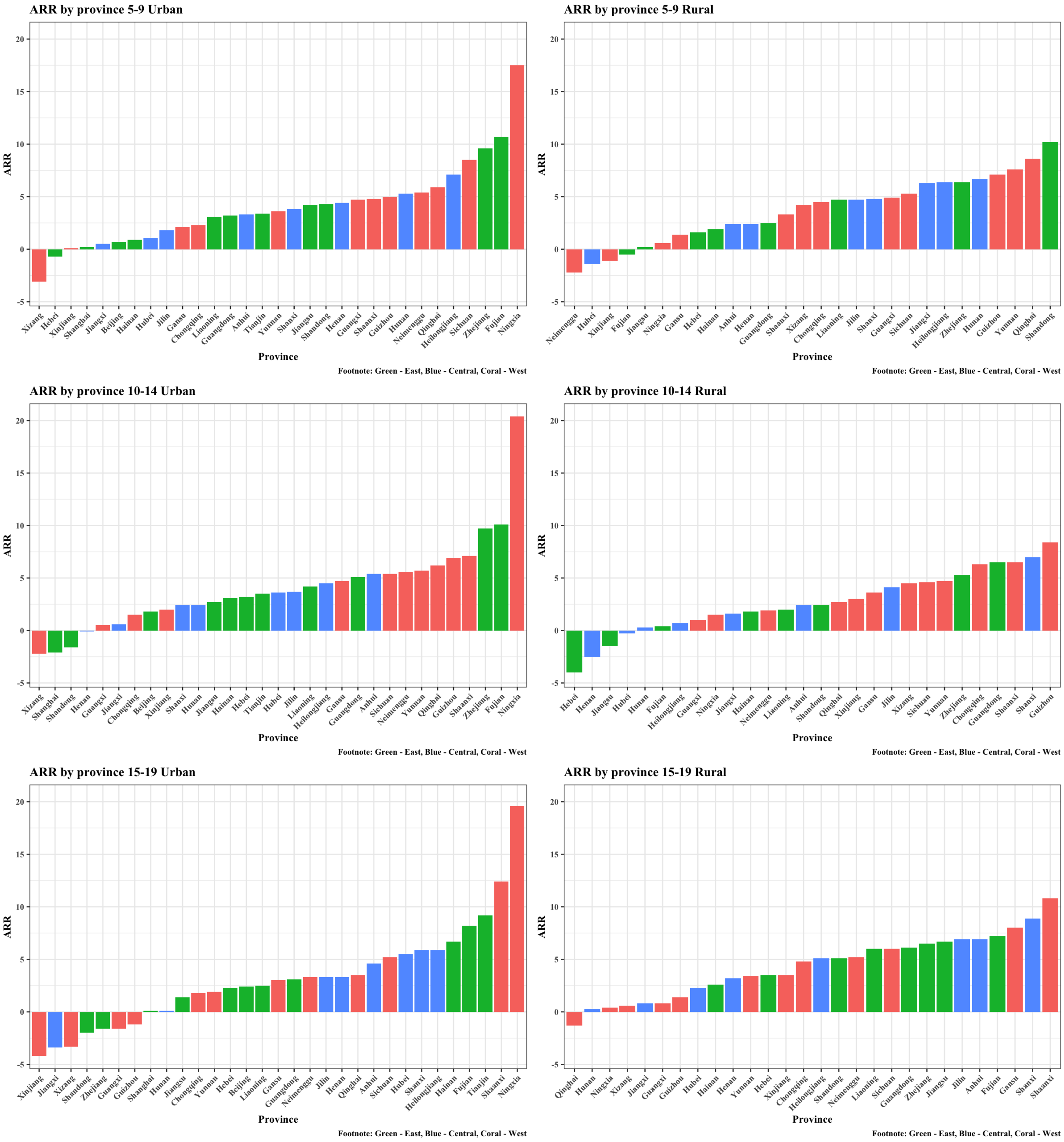

Figure S3. Provincial level ARR heatmaps

ARR heatmap, province, all causes

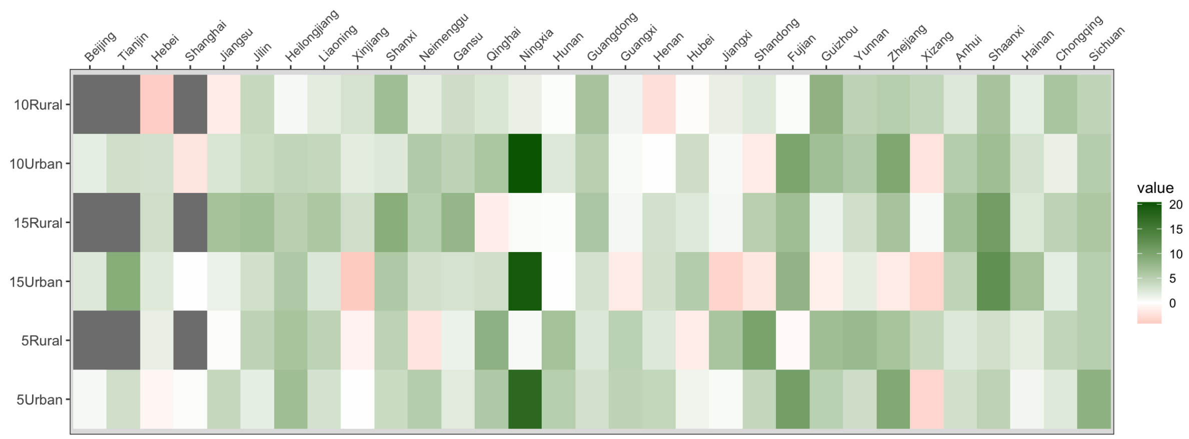

ARR heatmap, province, all causes

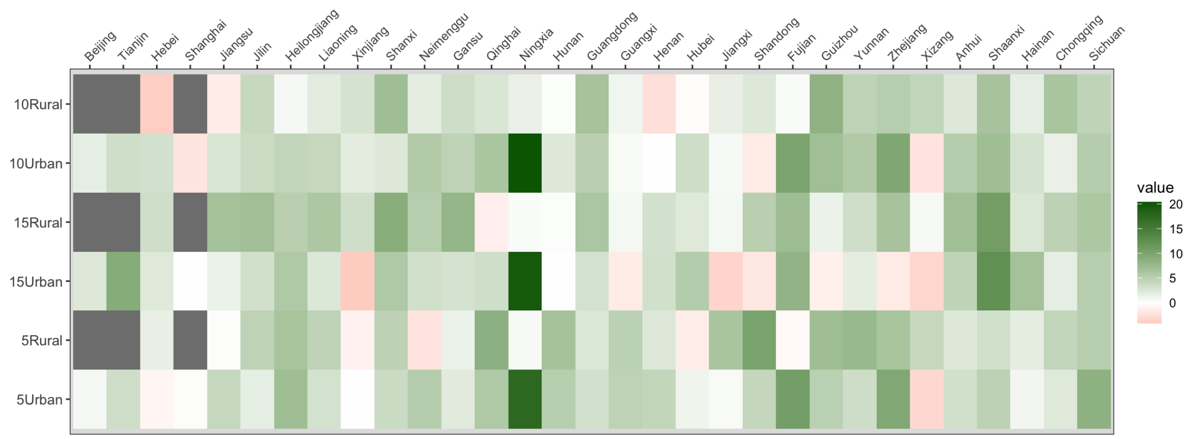

ARR heatmap, province, 5-9 Urban

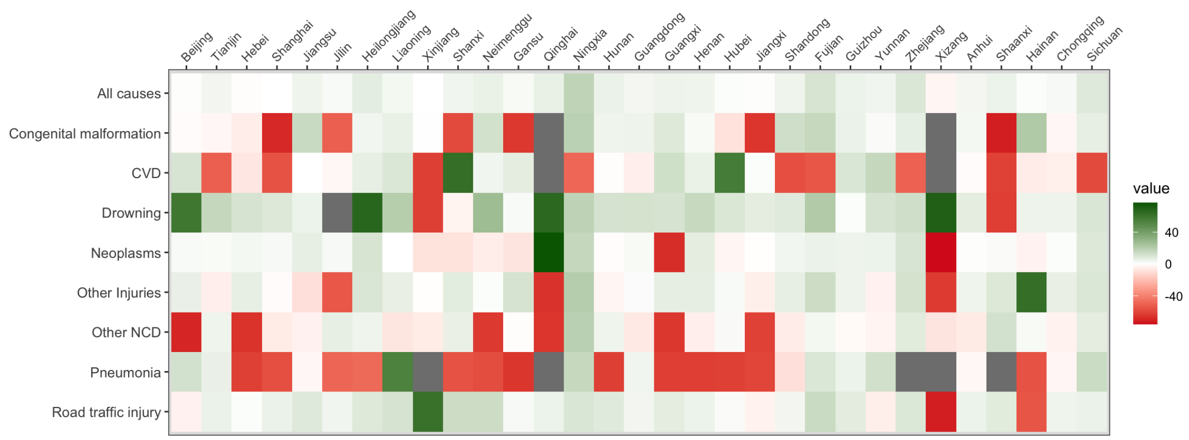

ARR heatmap, province, 5-9 Urban

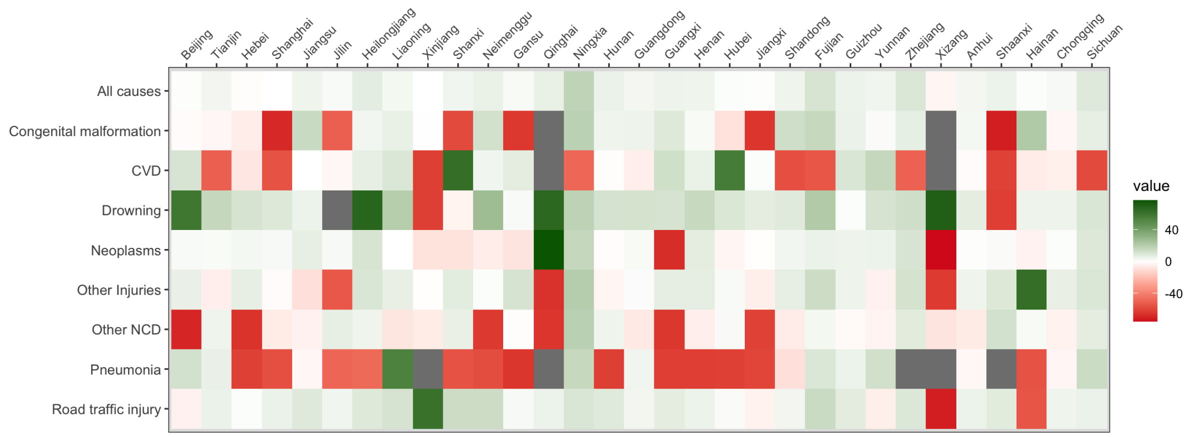

ARR heatmap, province, 5-9 Rural

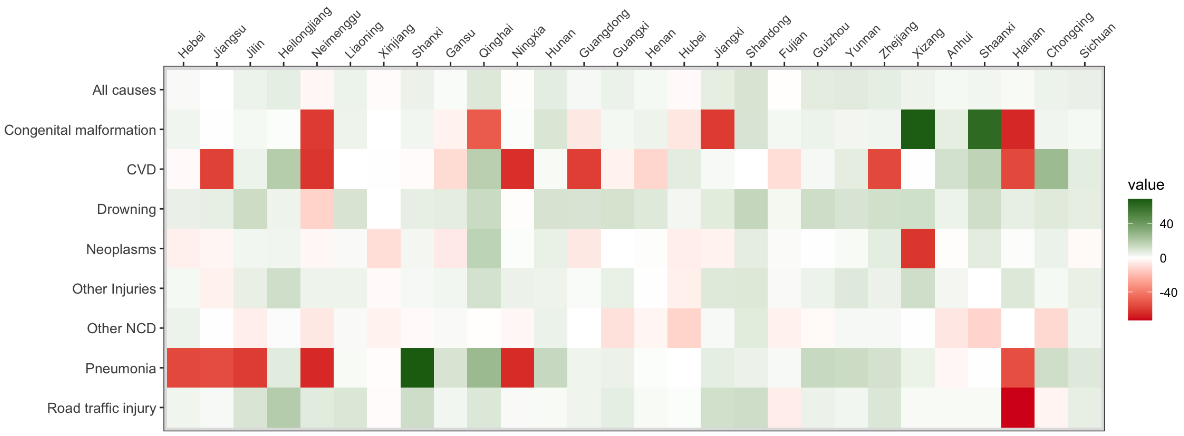

ARR heatmap, province, 5-9 Rural

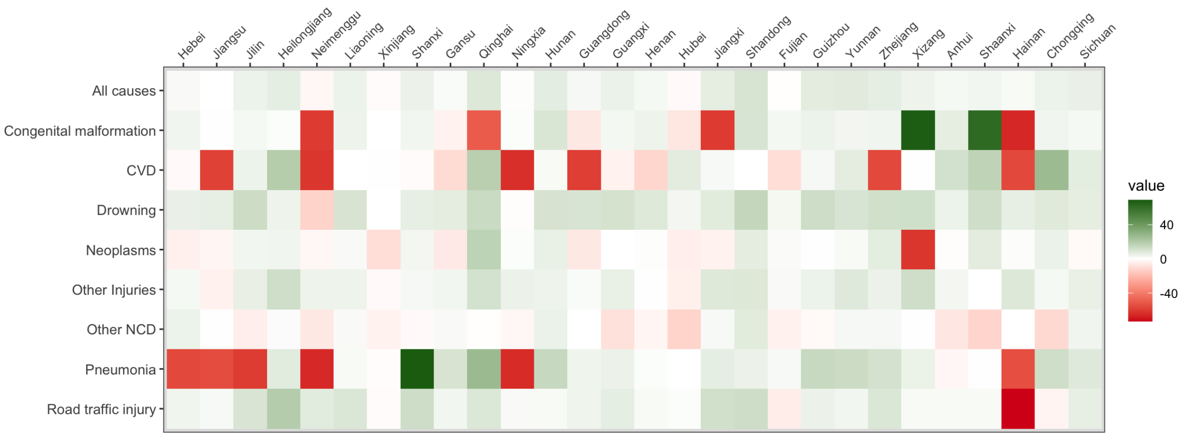

ARR heatmap, province, 10-14 Urban

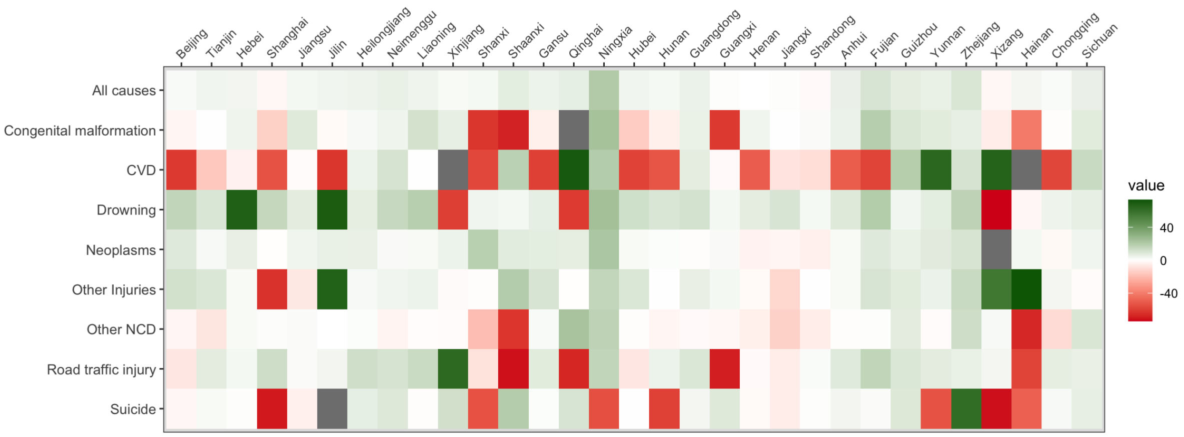

ARR heatmap, province, 10-14 Urban

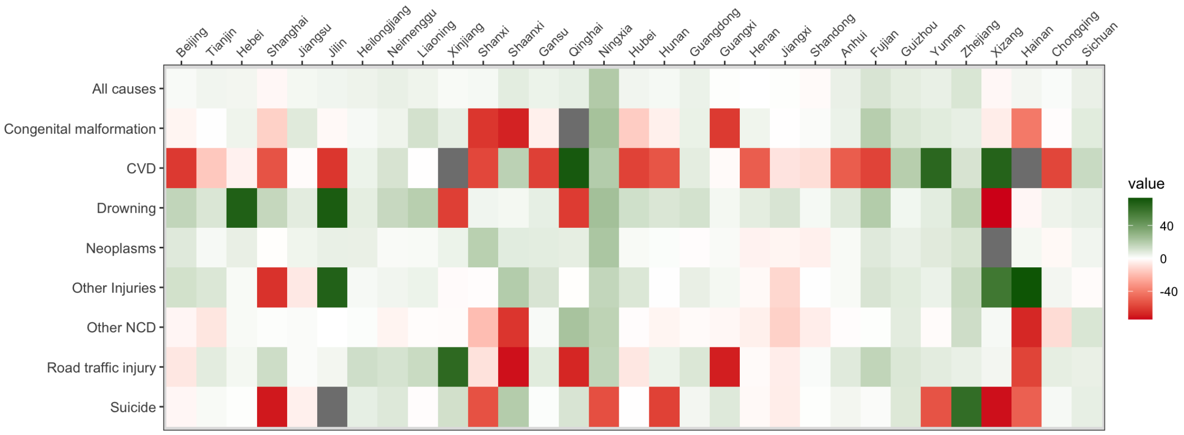

ARR heatmap, province, 10-14 Rural

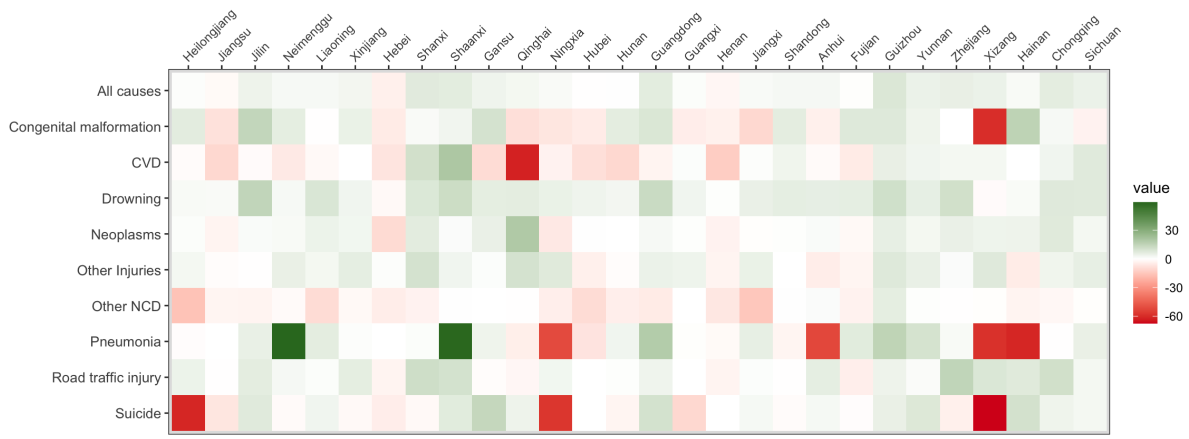

ARR heatmap, province, 10-14 Rural

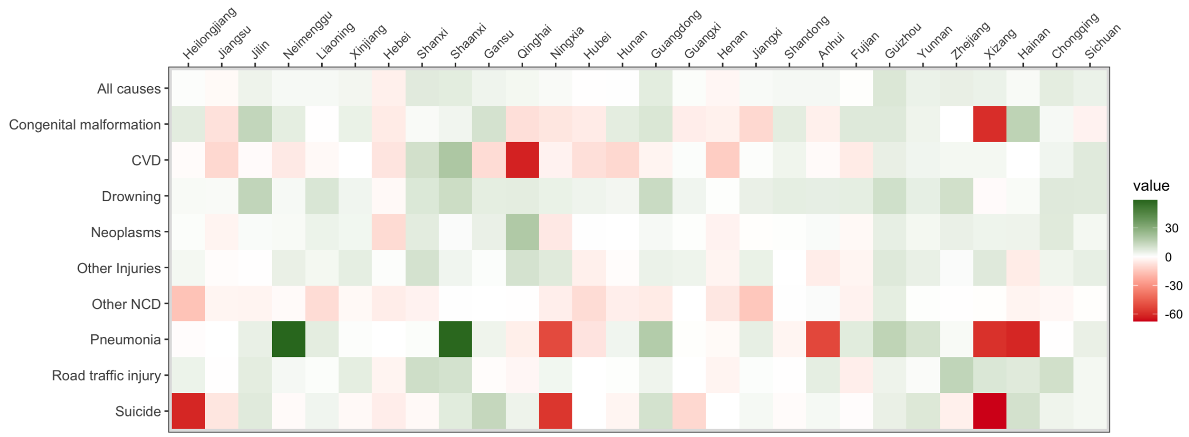

ARR heatmap, province, 15-19 Urban

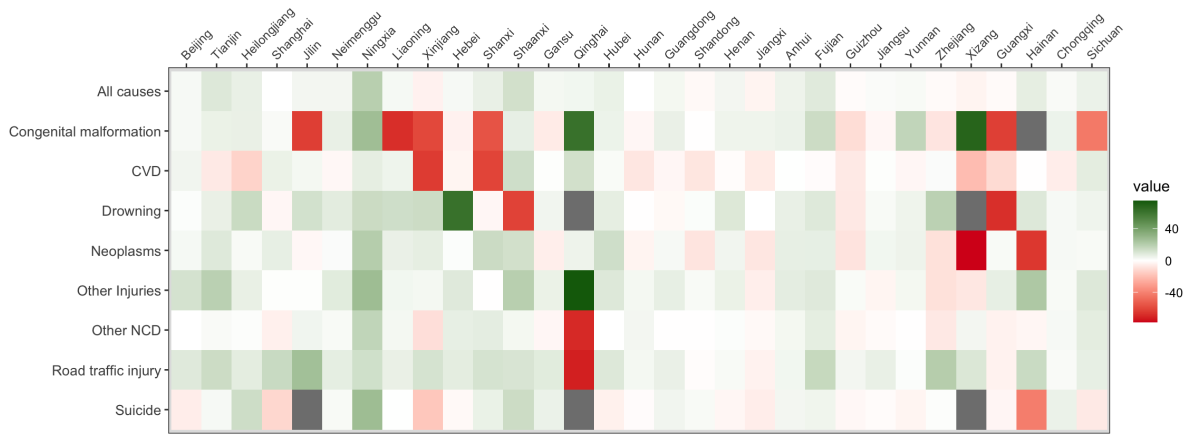

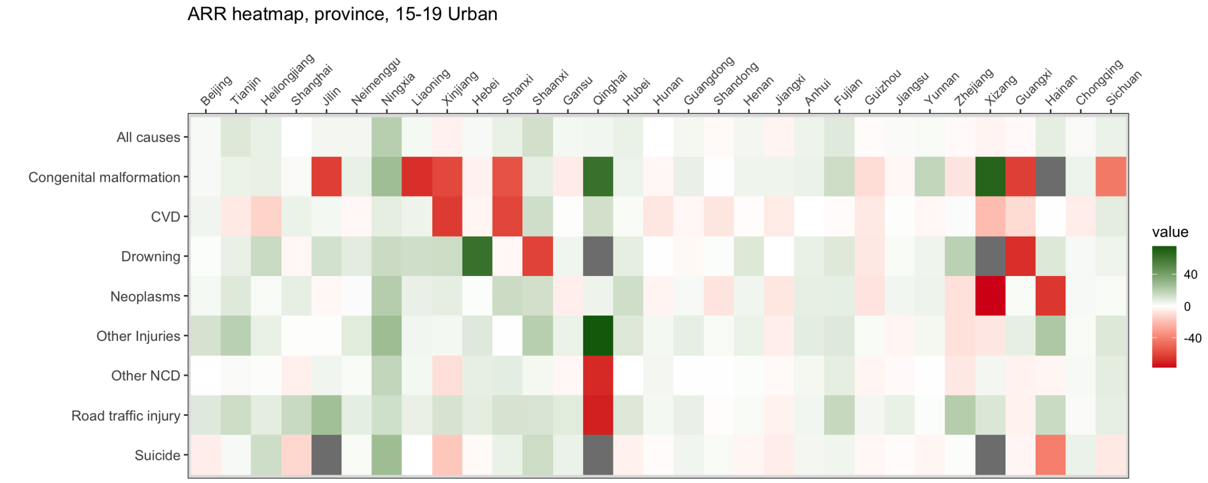

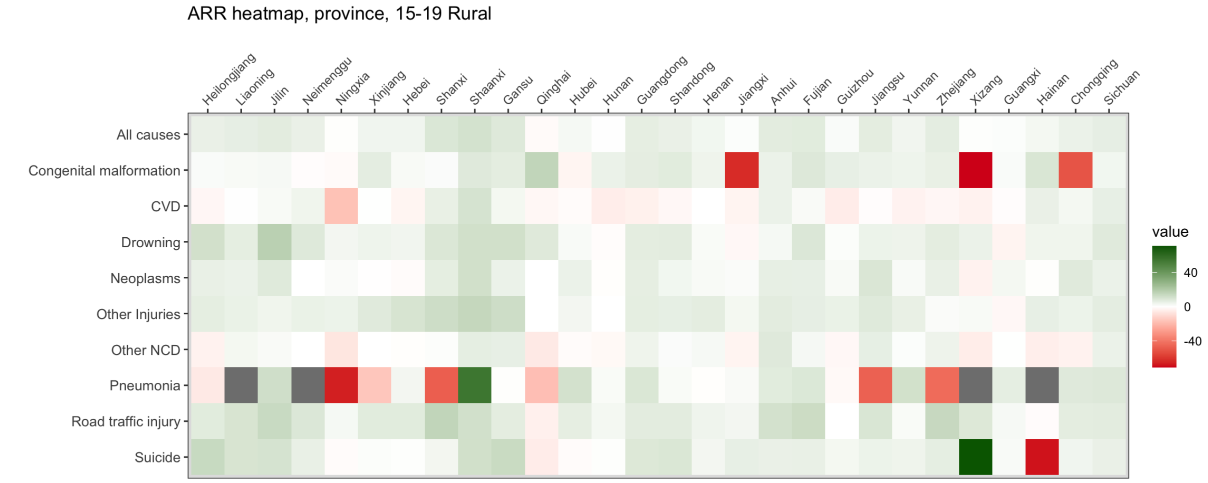

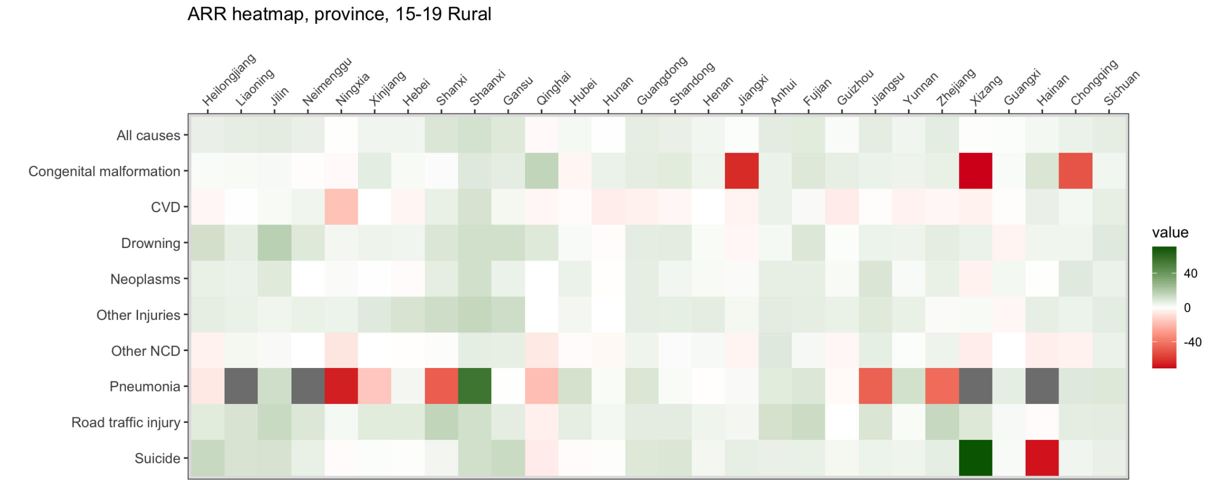

Figure S4. Strata trends of cause of deaths (COD) by age and gender in 2004-2019

Trend in causes of death, East Urban Both5-9 years, 2004-2019

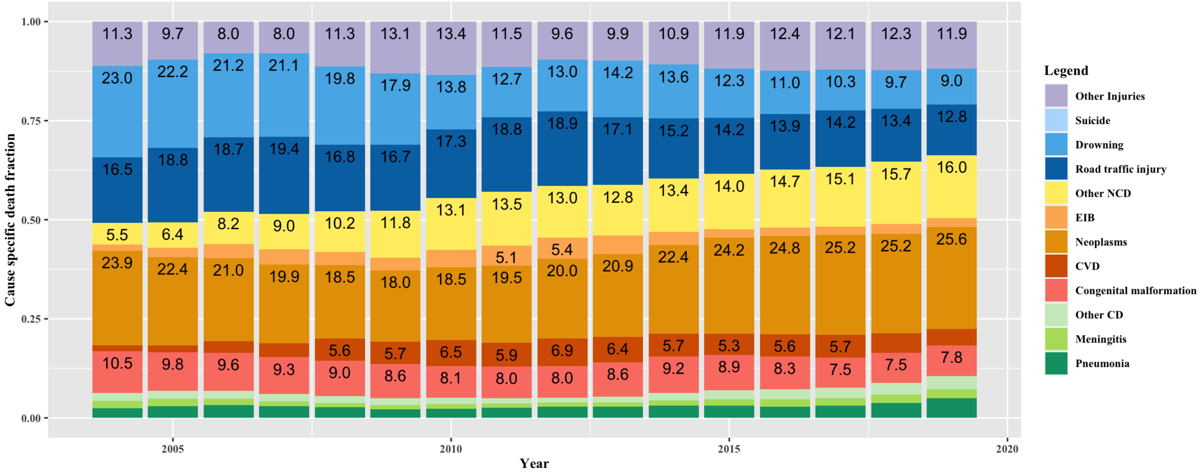

Trend in causes of death, East Urban Both10-14 years, 2004-2019

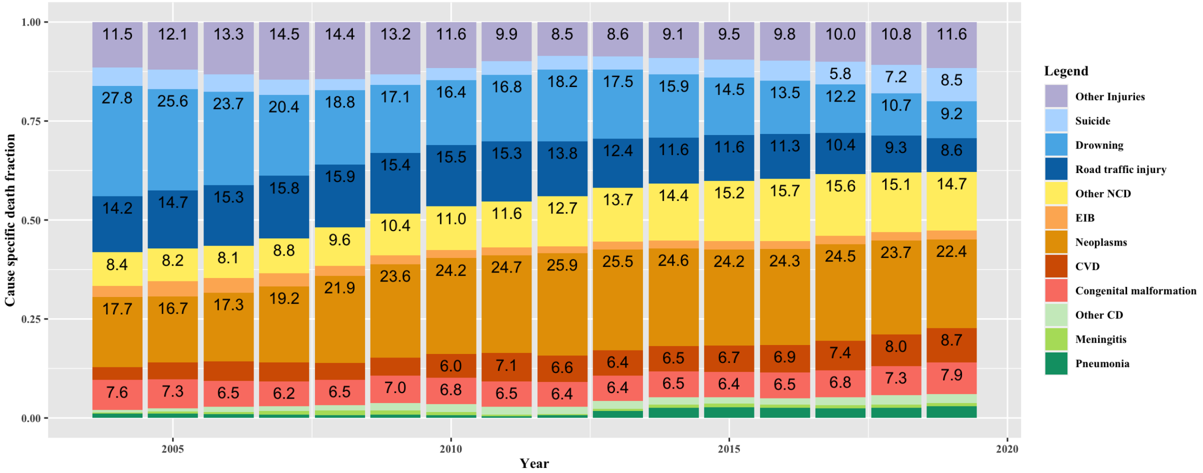

Trend in causes of death, East Urban Male15-19 years, 2004-2019

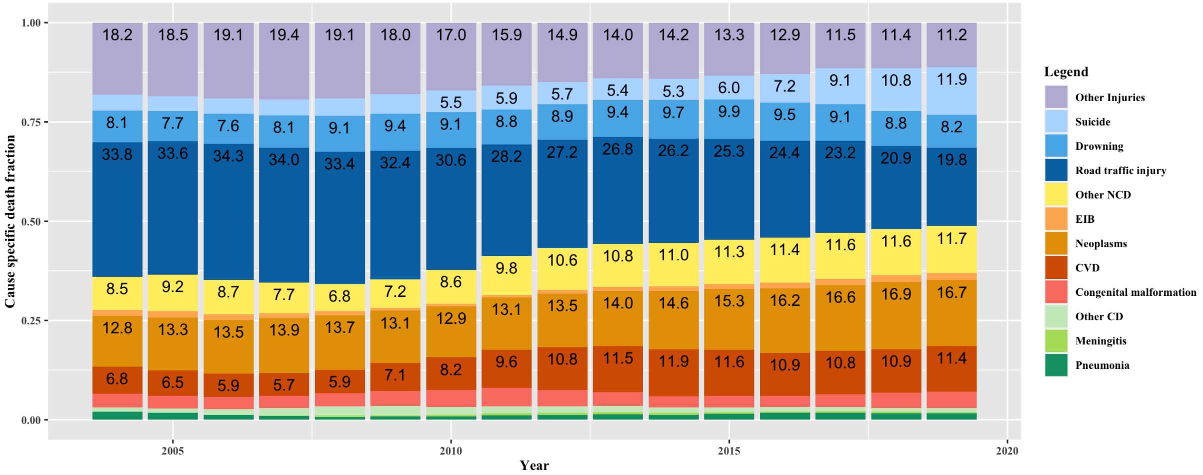

Trend in causes of death, East Urban Female15-19 years, 2004-2019

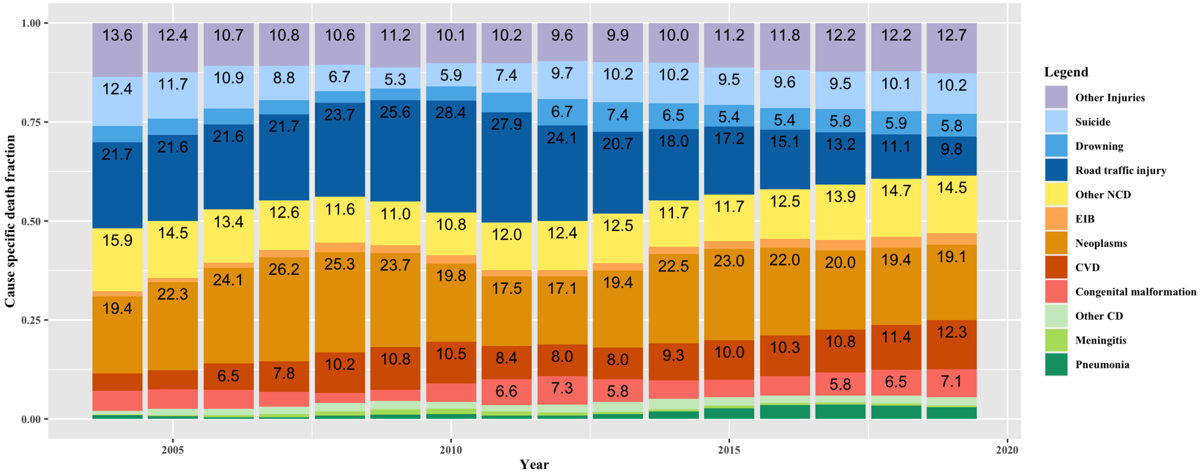

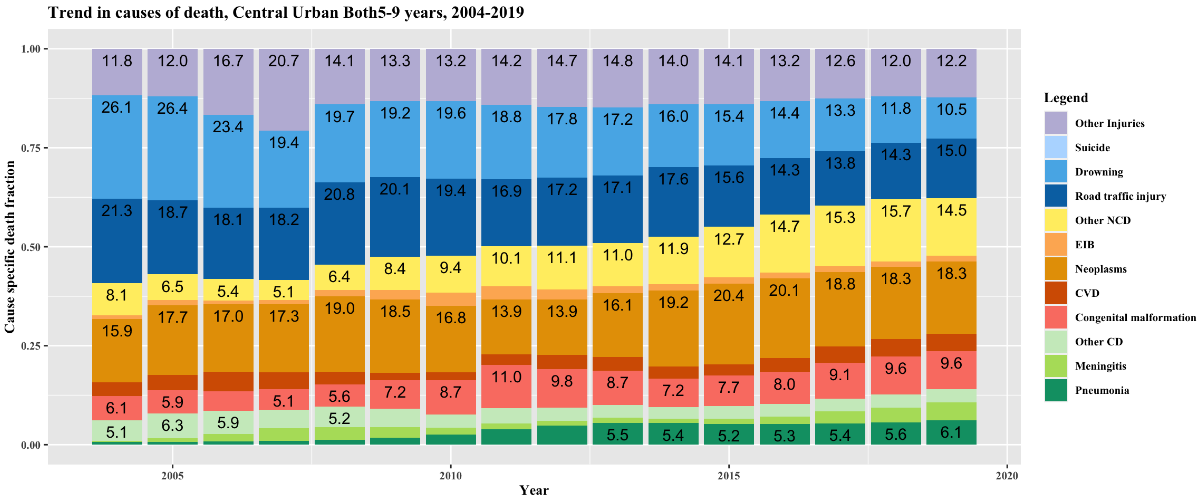
Trend in causes of death, Central Urban Both5-9 years, 2004-2019
Cause specific death fraction
Year
Legend
Other Injuries
Suicide
Drowning
Road traffic injury
Other NCD
EIB
Neoplasms
CVD
Congenital malformation
Other CD
Meningitis
Pneumonia

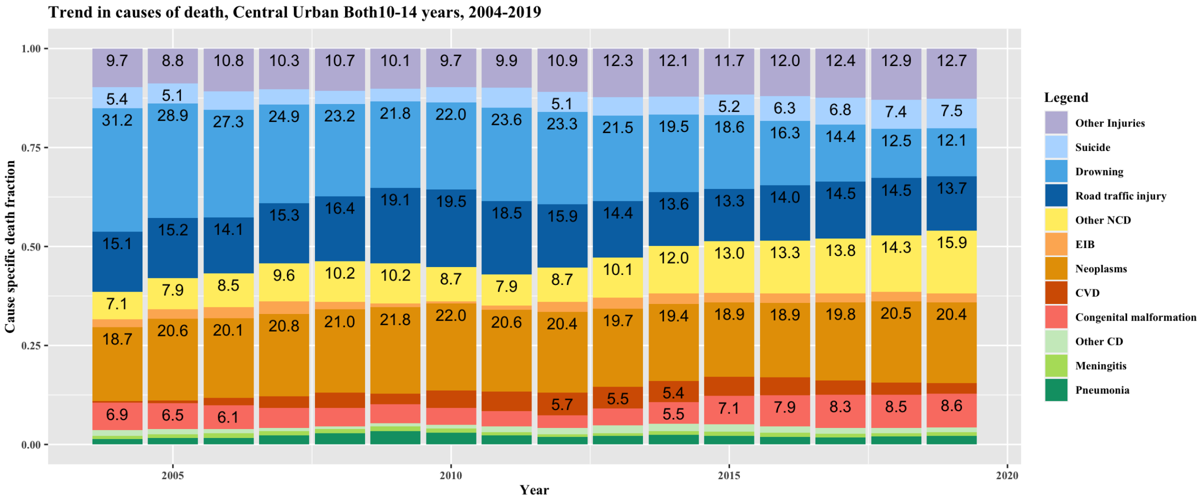
Trend in causes of death, Central Urban Both10-14 years, 2004-2019
Cause specific death fraction
Year
Legend
Other Injuries
Suicide
Drowning
Road traffic injury
Other NCD
EIB
Neoplasms
CVD
Congenital malformation
Other CD
Meningitis
Pneumonia

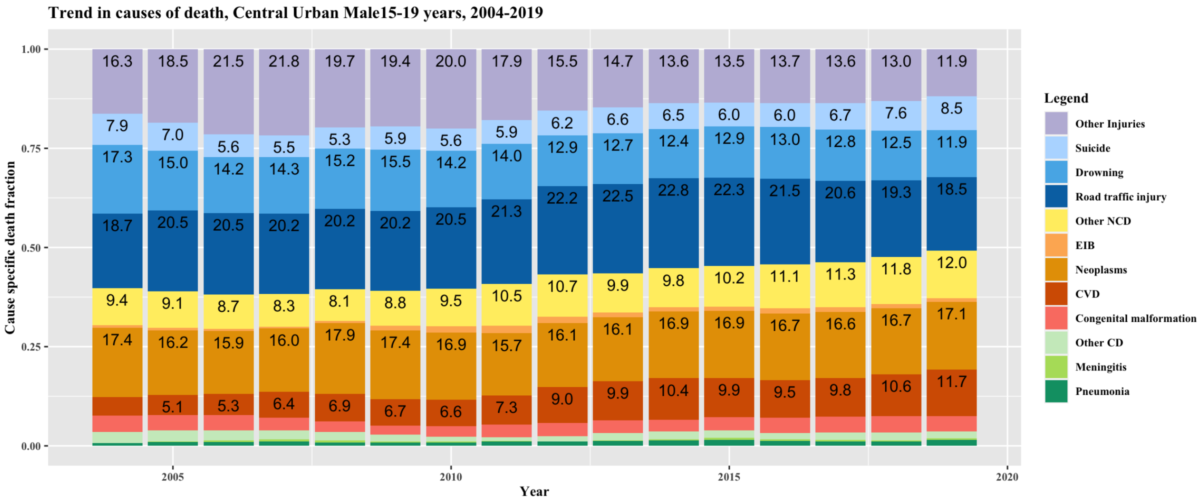
Trend in causes of death, Central Urban Male15-19 years, 2004-2019
Cause specific death fraction
Year
Legend
Other Injuries
Suicide
Drowning
Road traffic injury
Other NCD
EIB
Neoplasms
CVD
Congenital malformation
Other CD
Meningitis
Pneumonia

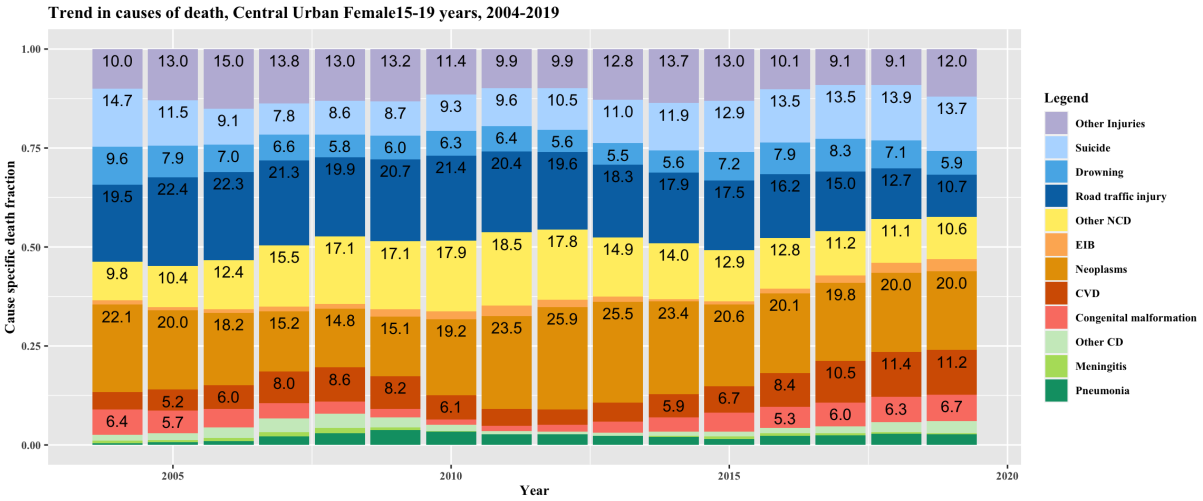
Trend in causes of death, Central Urban Female15-19 years, 2004-2019
Cause specific death fraction
Year
Legend
Other Injuries
Suicide
Drowning
Road traffic injury
Other NCD
EIB
Neoplasms
CVD
Congenital malformation
Other CD
Meningitis
Pneumonia

Trend in causes of death, West Urban Both5-9 years, 2004-2019

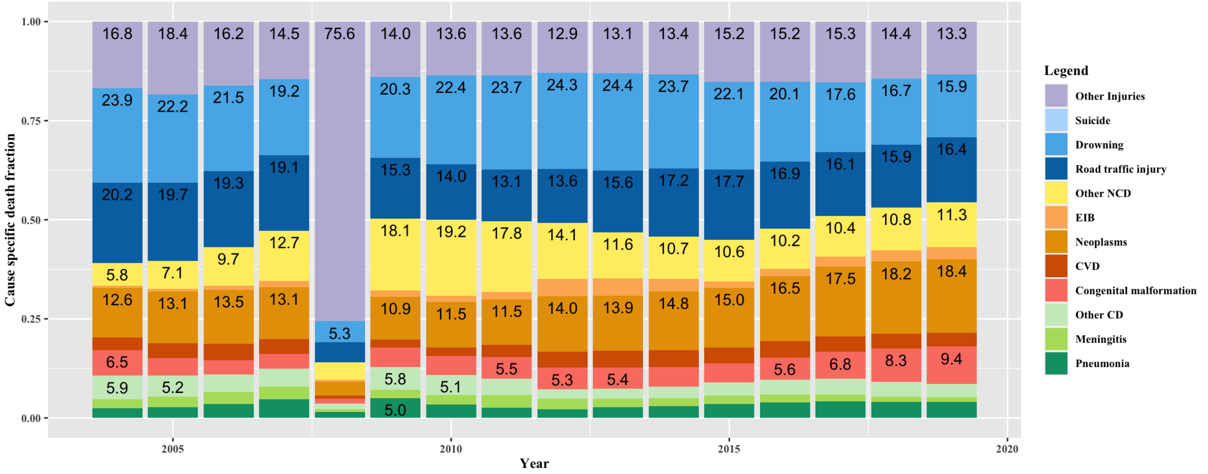

Trend in causes of death, West Urban Both10-14 years, 2004-2019

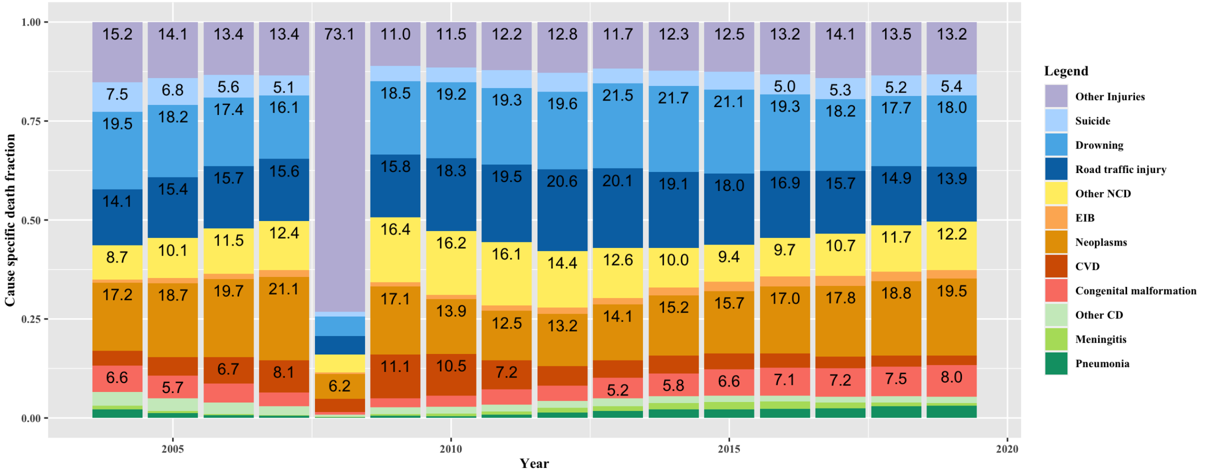

Trend in causes of death, West Urban Male15-19 years, 2004-2019

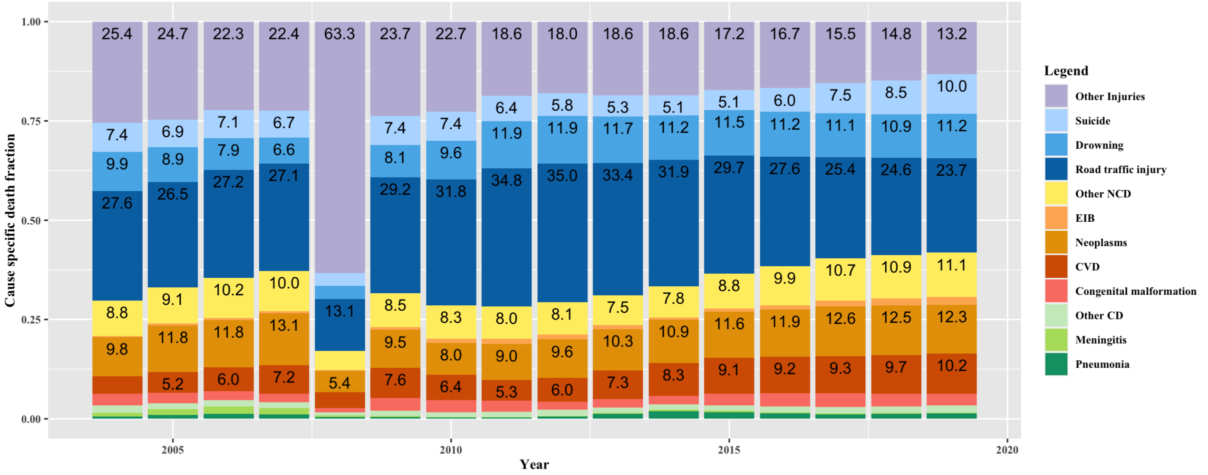

Trend in causes of death, West Urban Female15-19 years, 2004-2019

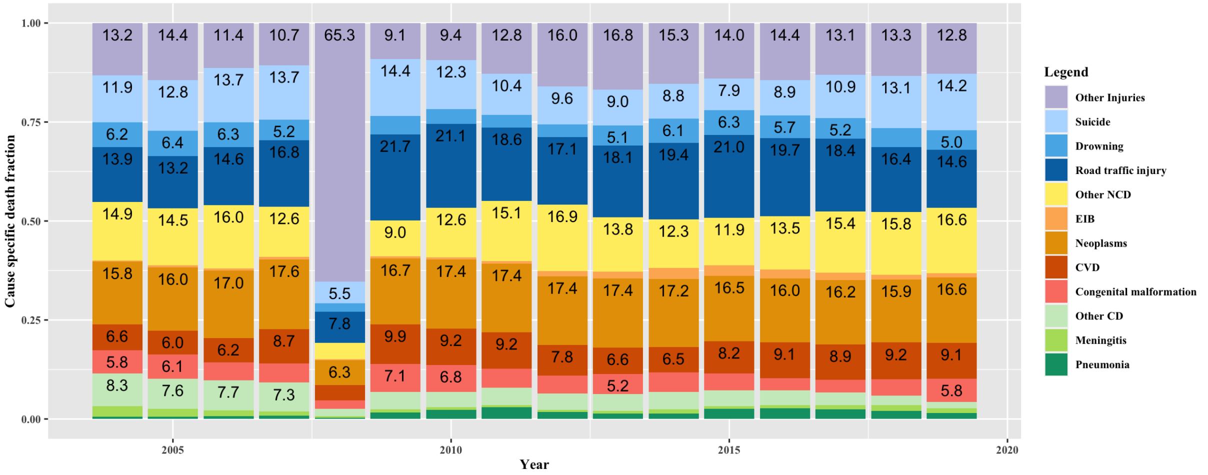

Trend in causes of death, East Rural Both5-9 years, 2004-2019

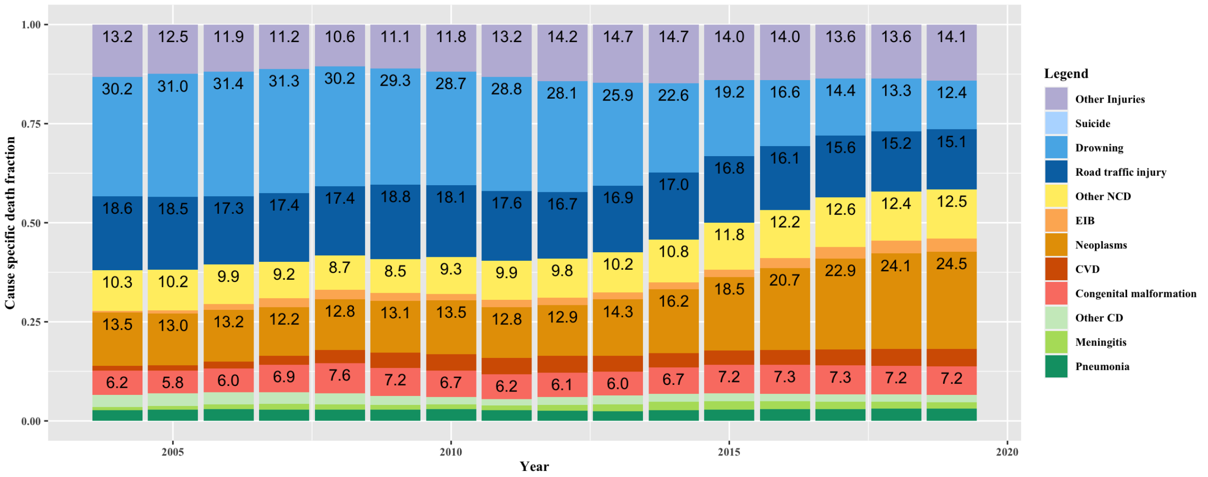

Trend in causes of death, East Rural Both10-14 years, 2004-2019

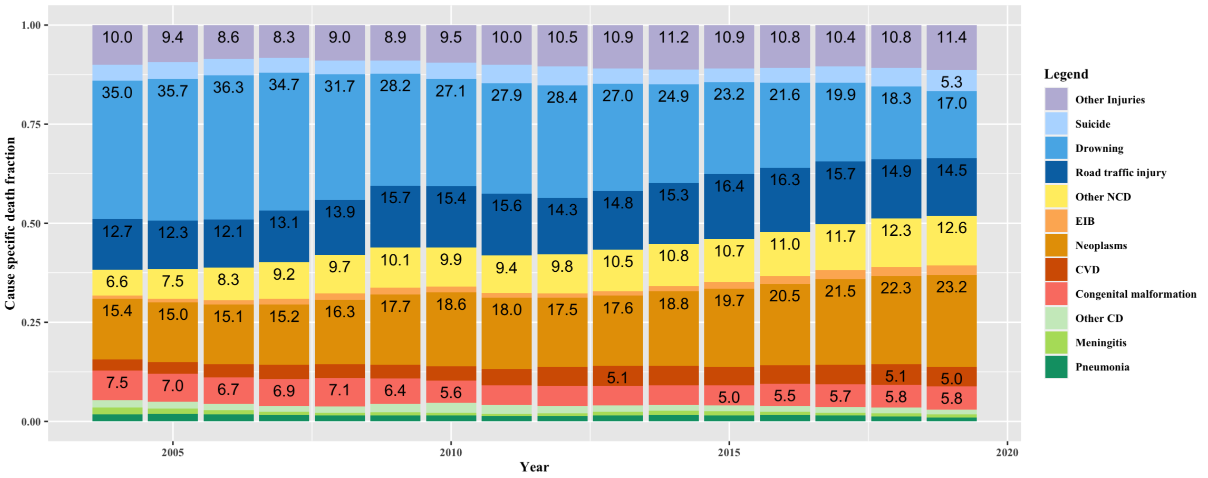

Trend in causes of death, East Rural Male15-19 years, 2004-2019

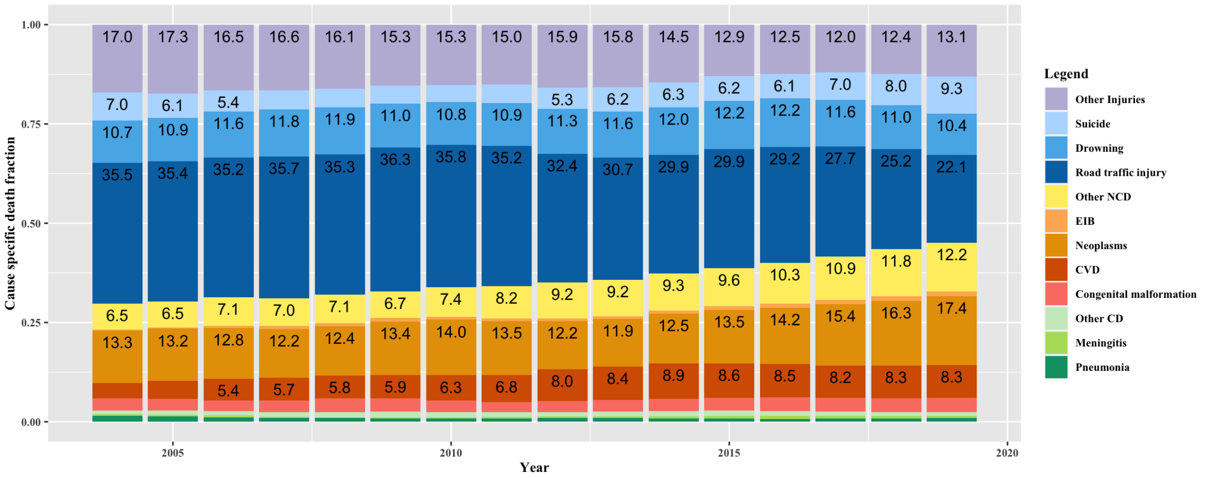

Trend in causes of death, East Rural Female15-19 years, 2004-2019

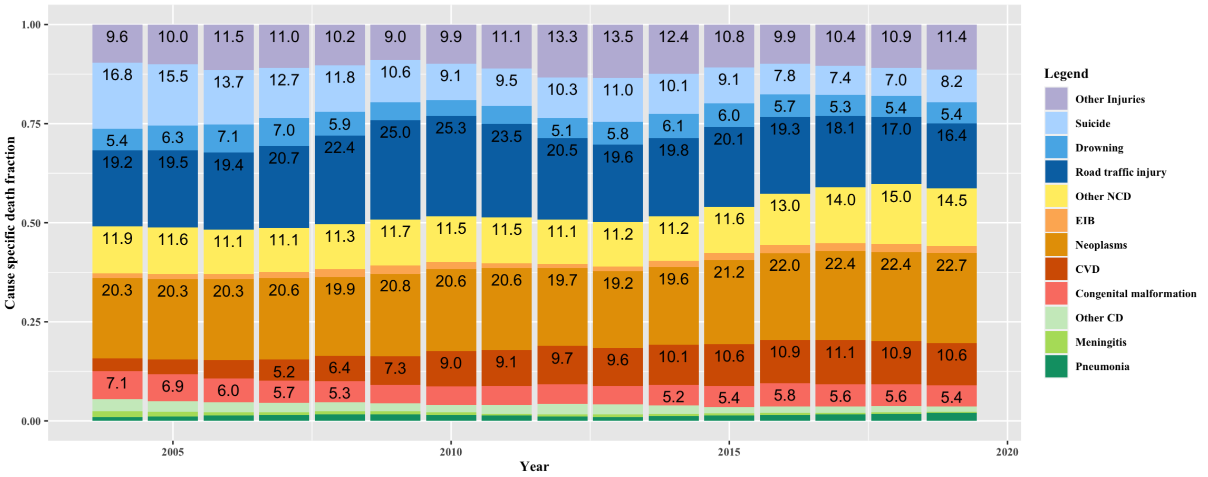

Trend in causes of death, Central Rural Both5-9 years, 2004-2019

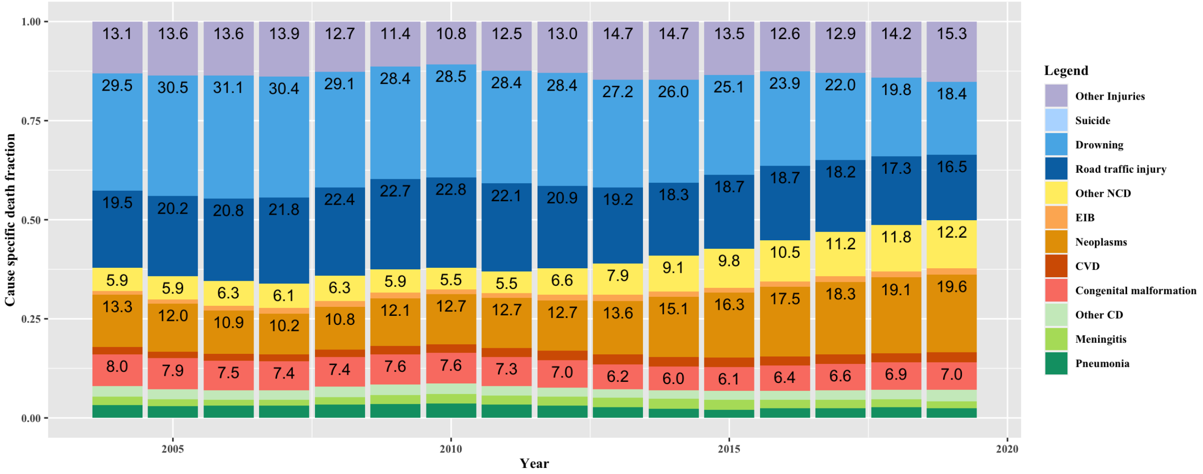

Trend in causes of death, Central Rural Both10-14 years, 2004-2019

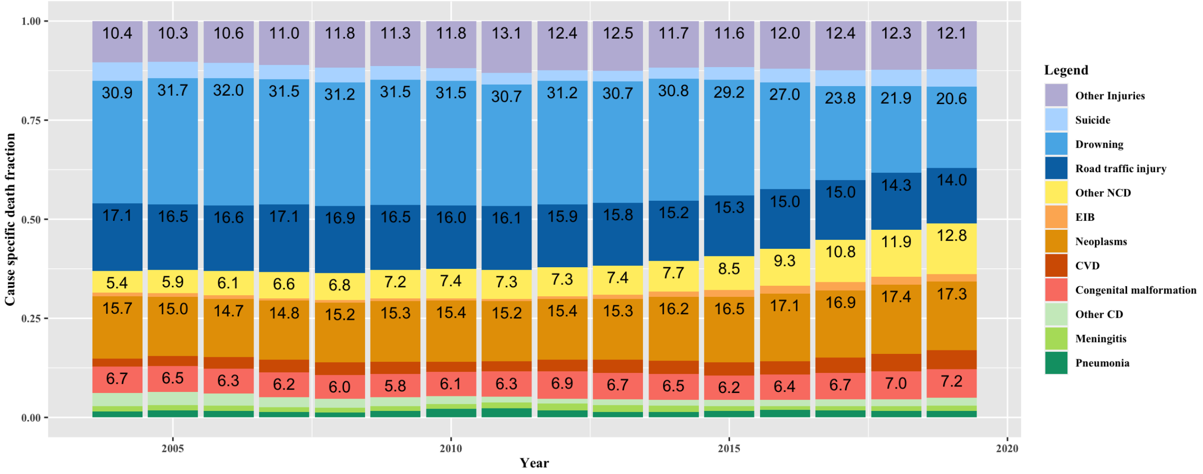

Trend in causes of death, Central Rural Male15-19 years, 2004-2019

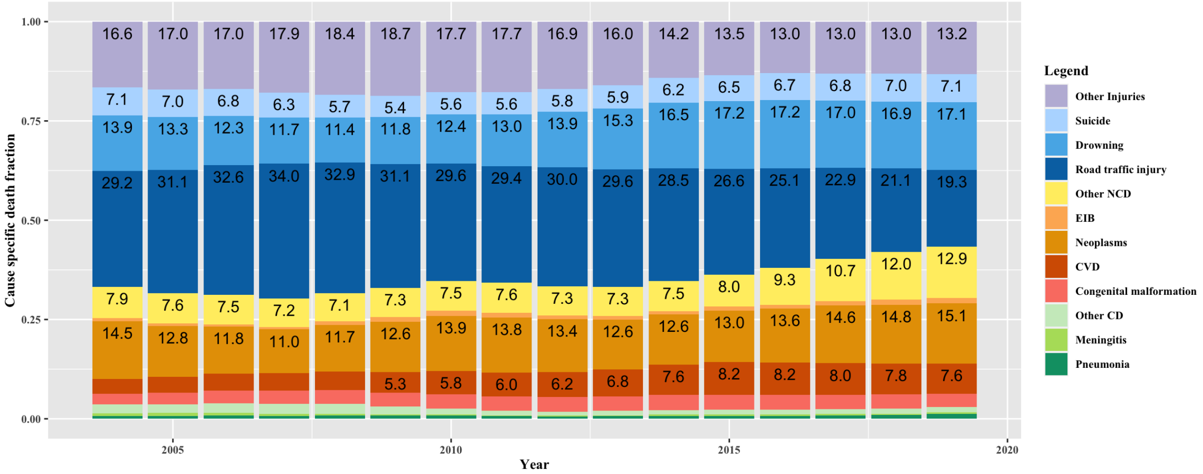

Trend in causes of death, Central Rural Female15-19 years, 2004-2019

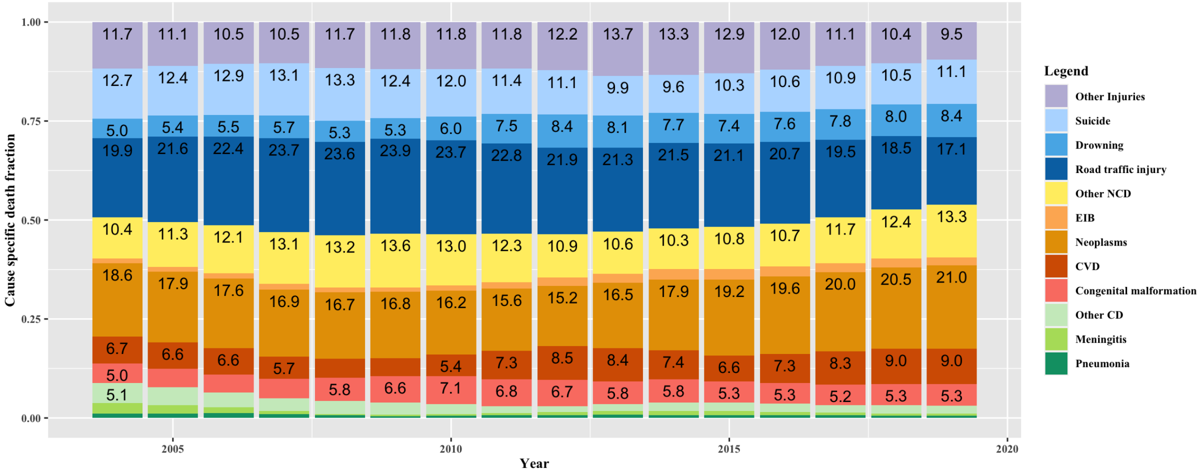

Trend in causes of death, West Rural Both5-9 years, 2004-2019

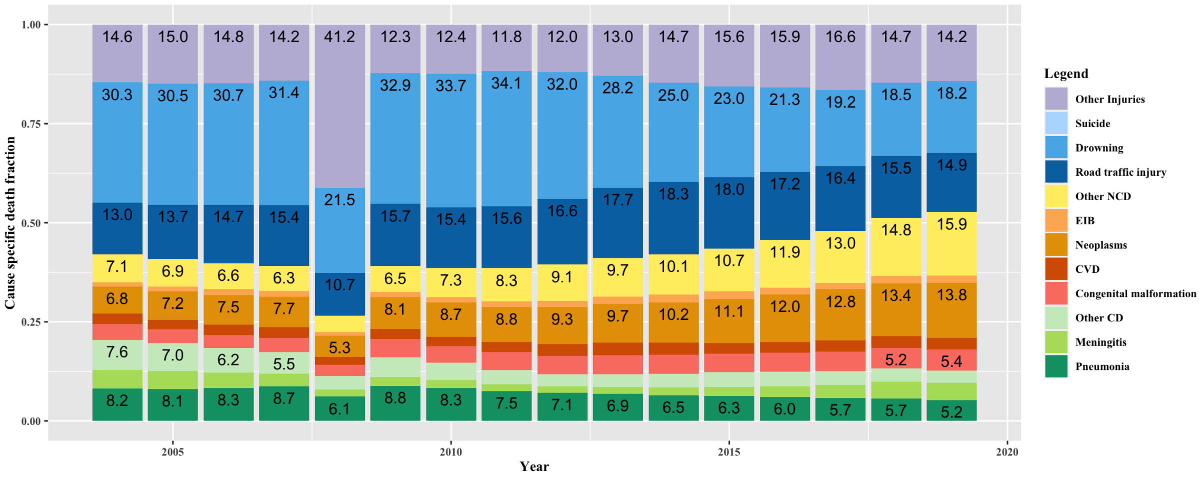

Trend in causes of death, West Rural Both10-14 years, 2004-2019

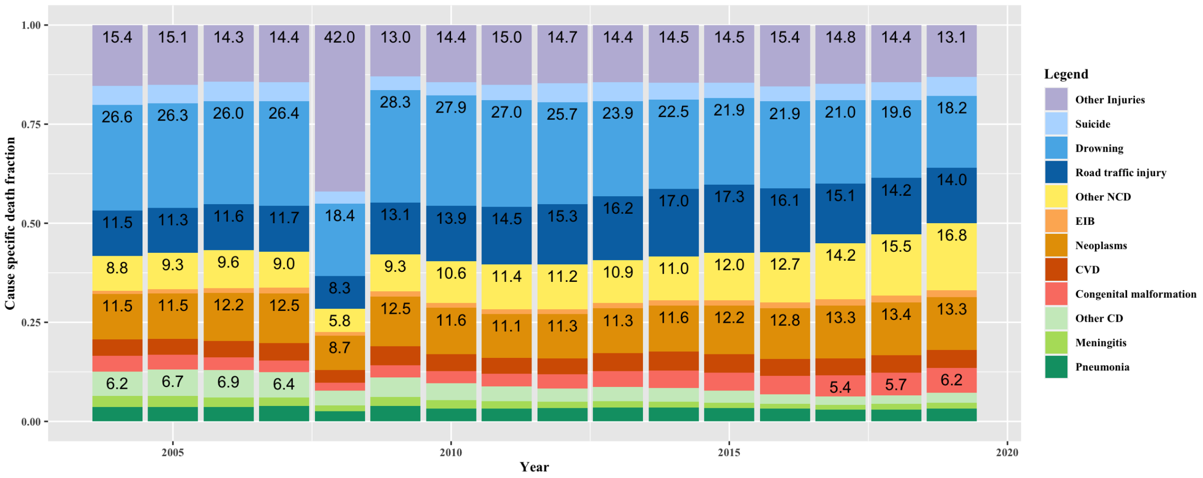

Trend in causes of death, West Rural Male15-19 years, 2004-2019

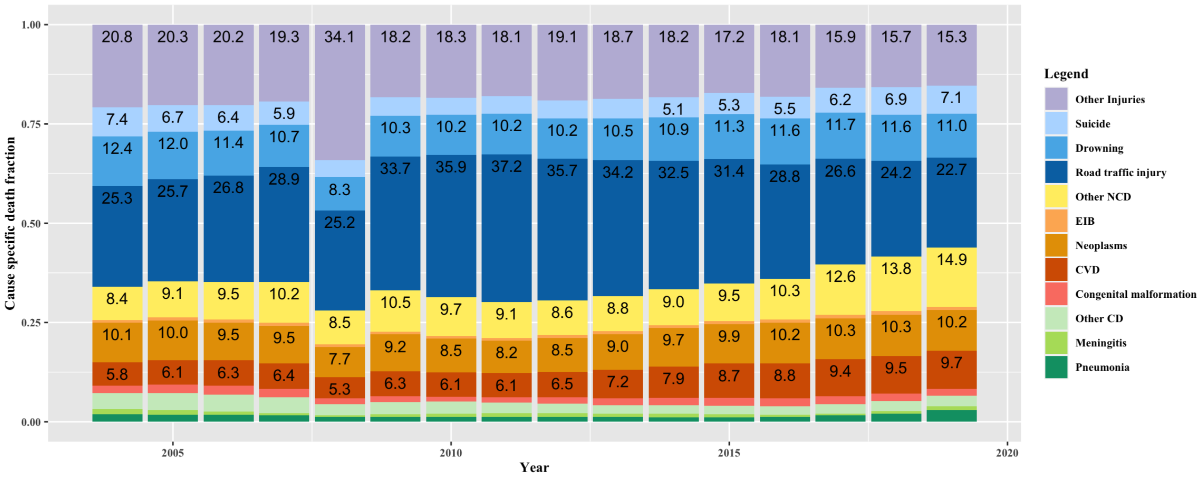

Trend in causes of death, West Rural Female15-19 years, 2004-2019

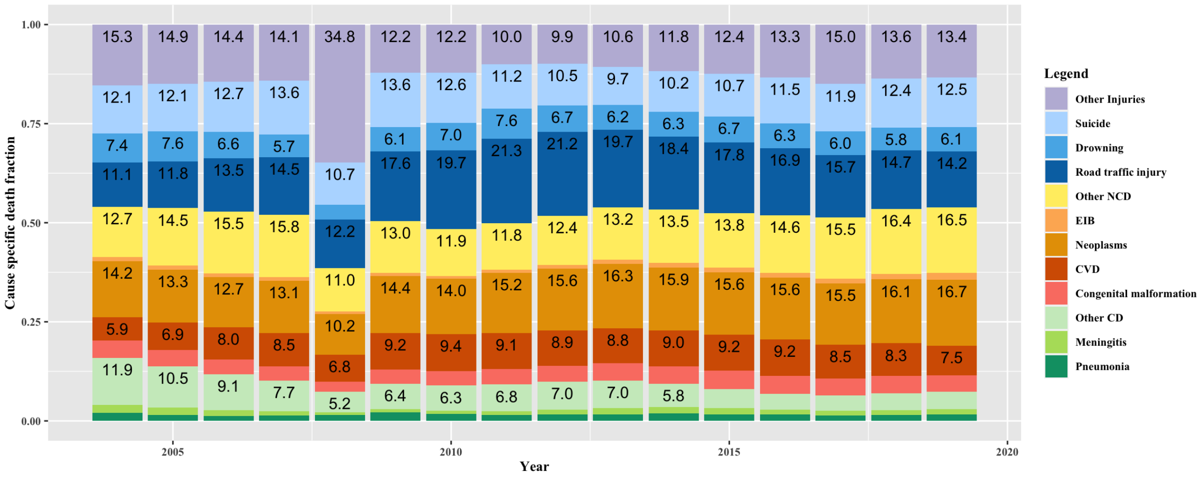

Figure S5. Province trends of cause of deaths (COD) in 2004-2019

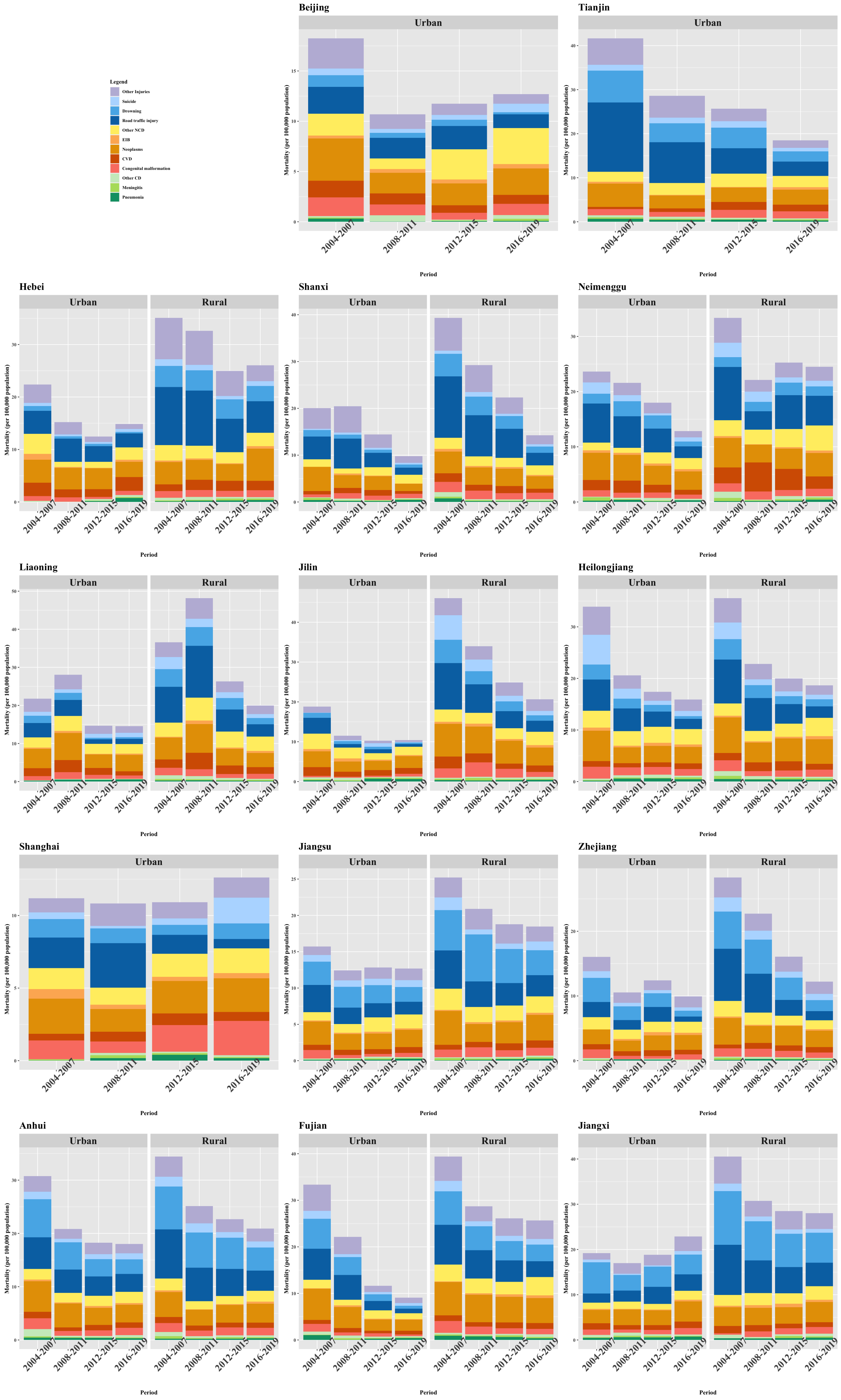
Legend
Other Injuries
Suicide
Drowning
Road traffic injury
Other NCD
EIB
Neoplasms
CVD
Congenital malformation
Other CD
Meningitis
Pneumonia
Beijing
Tianjin
Hebei
Shanxi
Neimenggu
Liaoning
Jilin
Heilongjiang
Shanghai
Jiangsu
Zhejiang
Anhui
Fujian
Jiangxi
Urban
Rural
Mortality (per 100,000 population)
Period
2004-2007
2008-2011
2012-2015
2016-2019

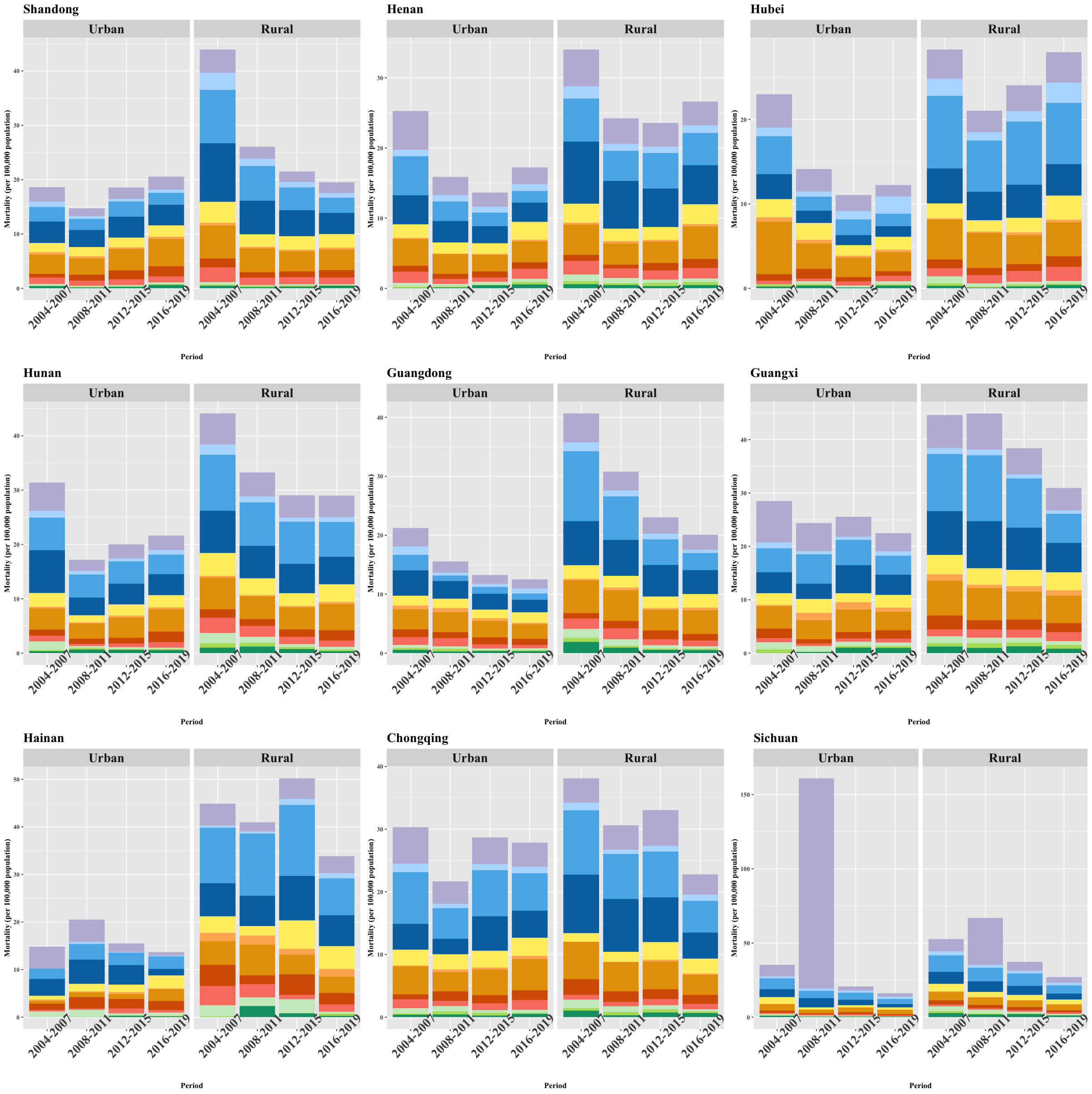
Shandong
Henan
Hubei
Hunan
Guangdong
Guangxi
Hainan
Chongqing
Sichuan
Urban
Rural
Mortality (per 100,000 population)
2004-2007
2008-2011
2012-2015
2016-2019
Period

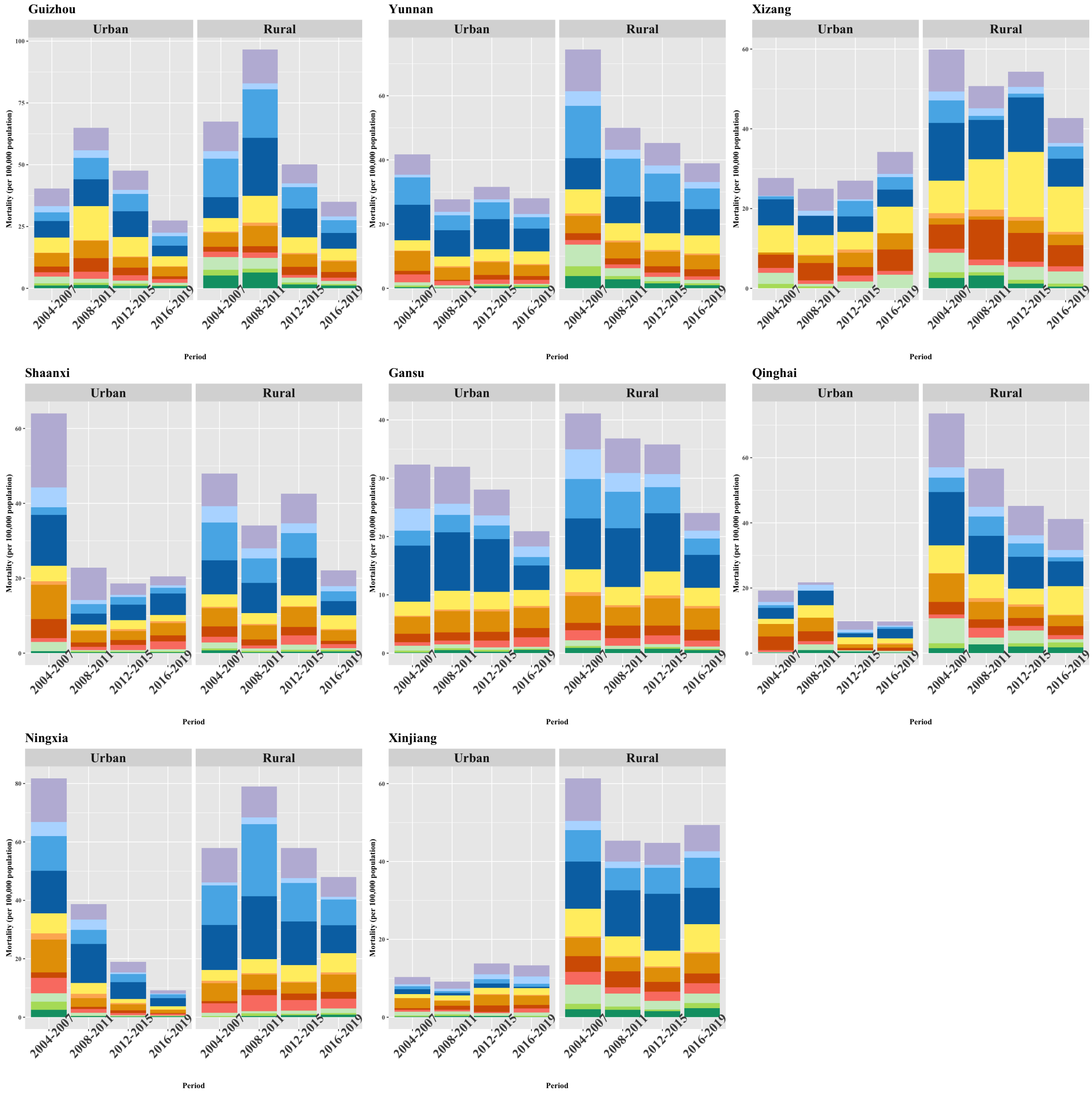
Guizhou
Yunnan
Xizang
Shaanxi
Gansu
Qinghai
Ningxia
Xinjiang
Urban
Rural
Mortality (per 100,000 population)
Period
2004-2007
2008-2011
2012-2015
2016-2019

Table S3. Region-residency strata level cause-specific death, fraction and mortality estimates, 2019

5-9 years							10-14 years							15-19 years				
Gender	Region	Residency	COD	CSND	CSDF	CSMR	Gender	Region	Residency	COD	CSND	CSDF	CSMR	Gender	Region	Residency	COD	CSND
Male	East	Urban	Neoplasms	263	0.27	3.5	Male	East	Urban	Neoplasms	215	0.23	2.9	Male	East	Urban	Road traffic injury	313
Male	East	Urban	Other NCD	133	0.14	1.8	Male	East	Urban	Drowning	121	0.13	1.6	Male	East	Urban	Neoplasms	263
Male	East	Urban	Road traffic injury	116	0.12	1.5	Male	East	Urban	Other NCD	110	0.11	1.5	Male	East	Urban	Suicide	188
Male	East	Urban	Other Injuries	114	0.12	1.5	Male	East	Urban	Other Injuries	98	0.1	1.3	Male	East	Urban	Other NCD	185
Male	East	Urban	Drowning	110	0.11	1.4	Male	East	Urban	Congenital malformation	90	0.09	1.2	Male	East	Urban	CVD	180
Male	East	Urban	Congenital malformation	73	0.08	1	Male	East	Urban	CVD	83	0.09	1.1	Male	East	Urban	Other Injuries	178
Male	East	Urban	Pneumonia	42	0.04	0.6	Male	East	Urban	Road traffic injury	82	0.09	1.1	Male	East	Urban	Drowning	130
Male	East	Urban	CVD	35	0.04	0.5	Male	East	Urban	Suicide	80	0.08	1.1	Male	East	Urban	Congenital malformation	66
Male	East	Urban	Other CD	29	0.03	0.4	Male	East	Urban	Pneumonia	26	0.03	0.4	Male	East	Urban	EIB	29
Male	East	Urban	EIB	•	•	•	Male	East	Urban	EIB	26	0.03	0.3	Male	East	Urban	Pneumonia	25
Male	East	Urban	Meningitis	•	•	•	Male	East	Urban	Other CD	•	•	•	Male	East	Urban	Other CD	
Male	East	Urban	Suicide	•	•	•	Male	East	Urban	Meningitis	•	•	•	Male	East	Urban	Meningitis	
Male	East	Rural	Neoplasms	483	0.25	4.8	Male	East	Rural	Neoplasms	466	0.23	5.4	Male	East	Rural	Road traffic injury	649
Male	East	Rural	Road traffic injury	301	0.16	3	Male	East	Rural	Drowning	426	0.21	4.9	Male	East	Rural	Neoplasms	510
Male	East	Rural	Drowning	279	0.14	2.8	Male	East	Rural	Road traffic injury	282	0.14	3.2	Male	East	Rural	Other Injuries	385
Male	East	Rural	Other Injuries	275	0.14	2.7	Male	East	Rural	Other NCD	244	0.12	2.8	Male	East	Rural	Other NCD	359
Male	East	Rural	Other NCD	216	0.11	2.2	Male	East	Rural	Other Injuries	222	0.11	2.6	Male	East	Rural	Drowning	304
Male	East	Rural	Congenital malformation	128	0.07	1.3	Male	East	Rural	Congenital malformation	124	0.06	1.4	Male	East	Rural	Suicide	274
Male	East	Rural	CVD	75	0.04	0.7	Male	East	Rural	Suicide	121	0.06	1.4	Male	East	Rural	CVD	244
Male	East	Rural	EIB	69	0.04	0.7	Male	East	Rural	CVD	95	0.05	1.1	Male	East	Rural	Congenital malformation	103
Male	East	Rural	Other CD	43	0.02	0.4	Male	East	Rural	EIB	35	0.02	0.4	Male	East	Rural	EIB	33
Male	East	Rural	Pneumonia	39	0.02	0.4	Male	East	Rural	Other CD	•	•	•	Male	East	Rural	Other CD	29
Male	East	Rural	Meningitis	31	0.02	0.3	Male	East	Rural	Pneumonia	•	•	•	Male	East	Rural	Pneumonia	27
Male	East	Rural	Suicide	•	•	•	Male	East	Rural	Meningitis	•	•	•	Male	East	Rural	Meningitis	
Male	Central	Urban	Neoplasms	99	0.16	2.6	Male	Central	Urban	Neoplasms	145	0.21	3.8	Male	Central	Urban	Road traffic injury	190
Male	Central	Urban	Road traffic injury	93	0.15	2.4	Male	Central	Urban	Other NCD	120	0.17	3.2	Male	Central	Urban	Neoplasms	176
Male	Central	Urban	Other NCD	91	0.15	2.4	Male	Central	Urban	Road traffic injury	104	0.15	2.7	Male	Central	Urban	Other NCD	124
Male	Central	Urban	Other Injuries	85	0.14	2.2	Male	Central	Urban	Drowning	104	0.15	2.7	Male	Central	Urban	Drowning	123
Male	Central	Urban	Drowning	74	0.12	2	Male	Central	Urban	Other Injuries	77	0.11	2	Male	Central	Urban	Other Injuries	122
Male	Central	Urban	Congenital malformation	64	0.1	1.7	Male	Central	Urban	Suicide	51	0.07	1.3	Male	Central	Urban	CVD	120
Male	Central	Urban	Pneumonia	35	0.06	0.9	Male	Central	Urban	Congenital malformation	50	0.07	1.3	Male	Central	Urban	Suicide	88
Male	Central	Urban	Meningitis	25	0.04	0.7	Male	Central	Urban	EIB	•	•	•	Male	Central	Urban	Congenital malformation	40
Male	Central	Urban	CVD	•	•	•	Male	Central	Urban	Pneumonia	•	•	•	Male	Central	Urban	Other CD	
Male	Central	Urban	Other CD	•	•	•	Male	Central	Urban	Other CD	•	•	•	Male	Central	Urban	Pneumonia	
Male	Central	Urban	EIB	•	•	•	Male	Central	Urban	CVD	•	•	•	Male	Central	Urban	EIB	
Male	Central	Urban	Suicide	•	•	•	Male	Central	Urban	Meningitis	•	•	•	Male	Central	Urban	Meningitis	
Male	Central	Rural	Drowning	534	0.22	4.4	Male	Central	Rural	Drowning	648	0.24	7.1	Male	Central	Rural	Road traffic injury	691
Male	Central	Rural	Neoplasms	462	0.19	3.8	Male	Central	Rural	Neoplasms	460	0.17	5.1	Male	Central	Rural	Drowning	611

Note:
Estimates are dashed-out if number of death is less than 25.

5-9 years							10-14 years							15-19 years				
Gender	Region	Residency	COD	CSND	CSDF	CSMR	Gender	Region	Residency	COD	CSND	CSDF	CSMR	Gender	Region	Residency	COD	CSND
Male	Central	Rural	Road traffic injury	381	0.15	3.1	Male	Central	Rural	Road traffic injury	379	0.14	4.2	Male	Central	Rural	Neoplasms	540
Male	Central	Rural	Other Injuries	376	0.15	3.1	Male	Central	Rural	Other Injuries	362	0.14	4	Male	Central	Rural	Other Injuries	471
Male	Central	Rural	Other NCD	283	0.11	2.3	Male	Central	Rural	Other NCD	325	0.12	3.6	Male	Central	Rural	Other NCD	461
Male	Central	Rural	Congenital malformation	163	0.07	1.3	Male	Central	Rural	Congenital malformation	139	0.05	1.5	Male	Central	Rural	CVD	273
Male	Central	Rural	Other CD	70	0.03	0.6	Male	Central	Rural	CVD	125	0.05	1.4	Male	Central	Rural	Suicide	255
Male	Central	Rural	CVD	62	0.02	0.5	Male	Central	Rural	Suicide	91	0.03	1	Male	Central	Rural	Congenital malformation	121
Male	Central	Rural	Pneumonia	58	0.02	0.5	Male	Central	Rural	Other CD	43	0.02	0.5	Male	Central	Rural	EIB	51
Male	Central	Rural	Meningitis	48	0.02	0.4	Male	Central	Rural	Meningitis	38	0.01	0.4	Male	Central	Rural	Other CD	48
Male	Central	Rural	EIB	42	0.02	0.3	Male	Central	Rural	EIB	36	0.01	0.4	Male	Central	Rural	Pneumonia	46
Male	Central	Rural	Suicide	•	•	•	Male	Central	Rural	Pneumonia	28	0.01	0.3	Male	Central	Rural	Meningitis	
Male	West	Urban	Drowning	161	0.2	3.9	Male	West	Urban	Drowning	178	0.21	3.6	Male	West	Urban	Road traffic injury	326
Male	West	Urban	Neoplasms	136	0.17	3.3	Male	West	Urban	Neoplasms	141	0.17	2.9	Male	West	Urban	Other Injuries	181
Male	West	Urban	Road traffic injury	120	0.15	2.9	Male	West	Urban	Road traffic injury	129	0.15	2.6	Male	West	Urban	Neoplasms	169
Male	West	Urban	Other Injuries	113	0.14	2.7	Male	West	Urban	Other Injuries	122	0.14	2.5	Male	West	Urban	Drowning	154
Male	West	Urban	Other NCD	99	0.12	2.4	Male	West	Urban	Other NCD	92	0.11	1.9	Male	West	Urban	Other NCD	153
Male	West	Urban	Congenital malformation	66	0.08	1.6	Male	West	Urban	Congenital malformation	60	0.07	1.2	Male	West	Urban	CVD	140
Male	West	Urban	Pneumonia	33	0.04	0.8	Male	West	Urban	Suicide	43	0.05	0.9	Male	West	Urban	Suicide	138
Male	West	Urban	Other CD	27	0.03	0.6	Male	West	Urban	Pneumonia	26	0.03	0.5	Male	West	Urban	Congenital malformation	40
Male	West	Urban	CVD	•	•	•	Male	West	Urban	EIB	•	•	•	Male	West	Urban	EIB	27
Male	West	Urban	EIB	•	•	•	Male	West	Urban	CVD	•	•	•	Male	West	Urban	Other CD	26
Male	West	Urban	Meningitis	•	•	•	Male	West	Urban	Other CD	•	•	•	Male	West	Urban	Pneumonia	
Male	West	Urban	Suicide	•	•	•	Male	West	Urban	Meningitis	•	•	•	Male	West	Urban	Meningitis	
Male	West	Rural	Drowning	662	0.22	7.6	Male	West	Rural	Drowning	609	0.22	5.5	Male	West	Rural	Road traffic injury	1075
Male	West	Rural	Road traffic injury	481	0.16	5.5	Male	West	Rural	Road traffic injury	471	0.17	4.3	Male	West	Rural	Other Injuries	725
Male	West	Rural	Other NCD	458	0.15	5.3	Male	West	Rural	Other NCD	420	0.15	3.8	Male	West	Rural	Other NCD	704
Male	West	Rural	Other Injuries	408	0.14	4.7	Male	West	Rural	Other Injuries	381	0.14	3.4	Male	West	Rural	Drowning	523
Male	West	Rural	Neoplasms	395	0.13	4.6	Male	West	Rural	Neoplasms	348	0.13	3.1	Male	West	Rural	Neoplasms	484
Male	West	Rural	Congenital malformation	135	0.04	1.6	Male	West	Rural	Congenital malformation	127	0.05	1.1	Male	West	Rural	CVD	459
Male	West	Rural	Pneumonia	127	0.04	1.5	Male	West	Rural	CVD	126	0.05	1.1	Male	West	Rural	Suicide	335
Male	West	Rural	Meningitis	114	0.04	1.3	Male	West	Rural	Suicide	99	0.04	0.9	Male	West	Rural	Pneumonia	138
Male	West	Rural	Other CD	93	0.03	1.1	Male	West	Rural	Pneumonia	64	0.02	0.6	Male	West	Rural	Other CD	129
Male	West	Rural	CVD	84	0.03	1	Male	West	Rural	Other CD	51	0.02	0.5	Male	West	Rural	Congenital malformation	78
Male	West	Rural	EIB	55	0.02	0.6	Male	West	Rural	Meningitis	44	0.02	0.4	Male	West	Rural	Meningitis	45
Male	West	Rural	Suicide	•	•	•	Male	West	Rural	EIB	44	0.02	0.4	Male	West	Rural	EIB	41
Female	East	Urban	Neoplasms	161	0.23	2.5	Female	East	Urban	Neoplasms	142	0.22	2.3	Female	East	Urban	Neoplasms	155
Female	East	Urban	Other NCD	131	0.19	2	Female	East	Urban	Other NCD	125	0.2	2.1	Female	East	Urban	Other NCD	117
Female	East	Urban	Road traffic injury	96	0.14	1.5	Female	East	Urban	Other Injuries	86	0.13	1.4	Female	East	Urban	Other Injuries	103
Female	East	Urban	Other Injuries	83	0.12	1.3	Female	East	Urban	CVD	56	0.09	0.9	Female	East	Urban	CVD	100
Female	East	Urban	Congenital malformation	56	0.08	0.9	Female	East	Urban	Suicide	56	0.09	0.9	Female	East	Urban	Suicide	83
Female	East	Urban	Pneumonia	40	0.06	0.6	Female	East	Urban	Road traffic injury	55	0.09	0.9	Female	East	Urban	Road traffic injury	80
Female	East	Urban	Drowning	39	0.06	0.6	Female	East	Urban	Congenital malformation	36	0.06	0.6	Female	East	Urban	Congenital malformation	58
Female	East	Urban	CVD	35	0.05	0.5	Female	East	Urban	Drowning	26	0.04	0.4	Female	East	Urban	Drowning	47

Note:
Estimates are dashed-out if number of death is less than 25.

5-9 years							10-14 years							15-19 years				
Gender	Region	Residency	COD	CSND	CSDF	CSMR	Gender	Region	Residency	COD	CSND	CSDF	CSMR	Gender	Region	Residency	COD	CSND
Female	East	Urban	Other CD	27	0.04	0.4	Female	East	Urban	Pneumonia	•	•	•	Female	East	Urban	Pneumonia	
Female	East	Urban	Meningitis	•	•	•	Female	East	Urban	Other CD	•	•	•	Female	East	Urban	EIB	
Female	East	Urban	EIB	•	•	•	Female	East	Urban	EIB	•	•	•	Female	East	Urban	Other CD	
Female	East	Urban	Suicide	•	•	•	Female	East	Urban	Meningitis	•	•	•	Female	East	Urban	Meningitis	
Female	East	Rural	Neoplasms	281	0.24	3.2	Female	East	Rural	Neoplasms	291	0.24	4	Female	East	Rural	Neoplasms	295
Female	East	Rural	Other NCD	174	0.15	2	Female	East	Rural	Road traffic injury	189	0.16	2.6	Female	East	Rural	Road traffic injury	213
Female	East	Rural	Road traffic injury	172	0.14	1.9	Female	East	Rural	Other NCD	165	0.14	2.3	Female	East	Rural	Other NCD	188
Female	East	Rural	Other Injuries	166	0.14	1.9	Female	East	Rural	Other Injuries	149	0.13	2	Female	East	Rural	Other Injuries	148
Female	East	Rural	Drowning	107	0.09	1.2	Female	East	Rural	Drowning	128	0.11	1.7	Female	East	Rural	CVD	138
Female	East	Rural	Congenital malformation	97	0.08	1.1	Female	East	Rural	CVD	68	0.06	0.9	Female	East	Rural	Suicide	106
Female	East	Rural	CVD	63	0.05	0.7	Female	East	Rural	Congenital malformation	64	0.05	0.9	Female	East	Rural	Congenital malformation	70
Female	East	Rural	Pneumonia	58	0.05	0.7	Female	East	Rural	Suicide	51	0.04	0.7	Female	East	Rural	Drowning	70
Female	East	Rural	EIB	34	0.03	0.4	Female	East	Rural	EIB	43	0.04	0.6	Female	East	Rural	Pneumonia	27
Female	East	Rural	Meningitis	•	•	•	Female	East	Rural	Other CD	•	•	•	Female	East	Rural	EIB	
Female	East	Rural	Other CD	•	•	•	Female	East	Rural	Meningitis	•	•	•	Female	East	Rural	Other CD	
Female	East	Rural	Suicide	•	•	•	Female	East	Rural	Pneumonia	•	•	•	Female	East	Rural	Meningitis	
Female	Central	Urban	Neoplasms	93	0.21	2.9	Female	Central	Urban	Neoplasms	73	0.2	2.3	Female	Central	Urban	Neoplasms	108
Female	Central	Urban	Road traffic injury	65	0.15	2	Female	Central	Urban	Other Injuries	58	0.16	1.8	Female	Central	Urban	Suicide	74
Female	Central	Urban	Other NCD	61	0.14	1.9	Female	Central	Urban	Other NCD	50	0.14	1.6	Female	Central	Urban	Other Injuries	65
Female	Central	Urban	Other Injuries	43	0.1	1.3	Female	Central	Urban	Road traffic injury	42	0.11	1.3	Female	Central	Urban	CVD	61
Female	Central	Urban	Congenital malformation	37	0.08	1.2	Female	Central	Urban	Congenital malformation	41	0.11	1.3	Female	Central	Urban	Road traffic injury	58
Female	Central	Urban	Drowning	36	0.08	1.1	Female	Central	Urban	Suicide	29	0.08	0.9	Female	Central	Urban	Other NCD	57
Female	Central	Urban	Pneumonia	29	0.07	0.9	Female	Central	Urban	Drowning	25	0.07	0.8	Female	Central	Urban	Congenital malformation	36
Female	Central	Urban	Meningitis	•	•	•	Female	Central	Urban	CVD	•	•	•	Female	Central	Urban	Drowning	32
Female	Central	Urban	Other CD	•	•	•	Female	Central	Urban	Pneumonia	•	•	•	Female	Central	Urban	Other CD	
Female	Central	Urban	CVD	•	•	•	Female	Central	Urban	EIB	•	•	•	Female	Central	Urban	EIB	
Female	Central	Urban	EIB	•	•	•	Female	Central	Urban	Other CD	•	•	•	Female	Central	Urban	Pneumonia	
Female	Central	Urban	Suicide	•	•	•	Female	Central	Urban	Meningitis	•	•	•	Female	Central	Urban	Meningitis	
Female	Central	Rural	Neoplasms	309	0.21	3.2	Female	Central	Rural	Neoplasms	268	0.17	3.6	Female	Central	Rural	Neoplasms	324
Female	Central	Rural	Road traffic injury	268	0.18	2.7	Female	Central	Rural	Drowning	220	0.14	2.9	Female	Central	Rural	Road traffic injury	263
Female	Central	Rural	Other Injuries	226	0.15	2.3	Female	Central	Rural	Other NCD	214	0.14	2.9	Female	Central	Rural	Other NCD	205
Female	Central	Rural	Other NCD	197	0.13	2	Female	Central	Rural	Road traffic injury	209	0.14	2.8	Female	Central	Rural	Suicide	171
Female	Central	Rural	Drowning	191	0.13	1.9	Female	Central	Rural	Congenital malformation	164	0.11	2.2	Female	Central	Rural	Other Injuries	146
Female	Central	Rural	Congenital malformation	112	0.08	1.1	Female	Central	Rural	Other Injuries	147	0.1	2	Female	Central	Rural	CVD	139
Female	Central	Rural	Other CD	40	0.03	0.4	Female	Central	Rural	Suicide	93	0.06	1.3	Female	Central	Rural	Drowning	130
Female	Central	Rural	Pneumonia	37	0.03	0.4	Female	Central	Rural	CVD	76	0.05	1	Female	Central	Rural	Congenital malformation	82
Female	Central	Rural	CVD	37	0.03	0.4	Female	Central	Rural	EIB	43	0.03	0.6	Female	Central	Rural	Other CD	32
Female	Central	Rural	Meningitis	•	•	•	Female	Central	Rural	Pneumonia	40	0.03	0.5	Female	Central	Rural	EIB	30
Female	Central	Rural	EIB	•	•	•	Female	Central	Rural	Other CD	38	0.02	0.5	Female	Central	Rural	Meningitis	
Female	Central	Rural	Suicide	•	•	•	Female	Central	Rural	Meningitis	•	•	•	Female	Central	Rural	Pneumonia	
Female	West	Urban	Neoplasms	113	0.2	2.9	Female	West	Urban	Neoplasms	128	0.24	2.9	Female	West	Urban	Neoplasms	99

Note:
Estimates are dashed-out if number of death is less than 25.

5-9 years							10-14 years							15-19 years				
Gender	Region	Residency	COD	CSND	CSDF	CSMR	Gender	Region	Residency	COD	CSND	CSDF	CSMR	Gender	Region	Residency	COD	CSND
Female	West	Urban	Road traffic injury	102	0.18	2.7	Female	West	Urban	Other NCD	77	0.15	1.7	Female	West	Urban	Other NCD	99
Female	West	Urban	Other Injuries	67	0.12	1.8	Female	West	Urban	Drowning	70	0.13	1.6	Female	West	Urban	Road traffic injury	87
Female	West	Urban	Congenital malformation	61	0.11	1.6	Female	West	Urban	Road traffic injury	64	0.12	1.4	Female	West	Urban	Suicide	85
Female	West	Urban	Drowning	54	0.1	1.4	Female	West	Urban	Other Injuries	61	0.11	1.4	Female	West	Urban	Other Injuries	77
Female	West	Urban	Other NCD	53	0.1	1.4	Female	West	Urban	Congenital malformation	51	0.1	1.1	Female	West	Urban	CVD	54
Female	West	Urban	EIB	29	0.05	0.8	Female	West	Urban	Suicide	31	0.06	0.7	Female	West	Urban	Congenital malformation	35
Female	West	Urban	CVD	26	0.05	0.7	Female	West	Urban	Pneumonia	•	•	•	Female	West	Urban	Drowning	30
Female	West	Urban	Pneumonia	•	•	•	Female	West	Urban	CVD	•	•	•	Female	West	Urban	Other CD	
Female	West	Urban	Other CD	•	•	•	Female	West	Urban	EIB	•	•	•	Female	West	Urban	Pneumonia	
Female	West	Urban	Meningitis	•	•	•	Female	West	Urban	Meningitis	•	•	•	Female	West	Urban	Meningitis	
Female	West	Urban	Suicide	•	•	•	Female	West	Urban	Other CD	•	•	•	Female	West	Urban	EIB	
Female	West	Rural	Other NCD	344	0.17	4.3	Female	West	Rural	Other NCD	355	0.2	3.5	Female	West	Rural	Neoplasms	383
Female	West	Rural	Other Injuries	309	0.15	3.8	Female	West	Rural	Neoplasms	264	0.15	2.6	Female	West	Rural	Other NCD	378
Female	West	Rural	Neoplasms	302	0.15	3.7	Female	West	Rural	Drowning	229	0.13	2.2	Female	West	Rural	Road traffic injury	326
Female	West	Rural	Road traffic injury	272	0.13	3.4	Female	West	Rural	Other Injuries	224	0.12	2.2	Female	West	Rural	Other Injuries	307
Female	West	Rural	Drowning	255	0.13	3.2	Female	West	Rural	Road traffic injury	171	0.09	1.7	Female	West	Rural	Suicide	286
Female	West	Rural	Pneumonia	135	0.07	1.7	Female	West	Rural	Congenital malformation	156	0.09	1.5	Female	West	Rural	CVD	172
Female	West	Rural	Congenital malformation	134	0.07	1.7	Female	West	Rural	Suicide	117	0.06	1.1	Female	West	Rural	Drowning	140
Female	West	Rural	Meningitis	110	0.05	1.4	Female	West	Rural	CVD	85	0.05	0.8	Female	West	Rural	Other CD	102
Female	West	Rural	Other CD	62	0.03	0.8	Female	West	Rural	Pneumonia	84	0.05	0.8	Female	West	Rural	Congenital malformation	94
Female	West	Rural	CVD	62	0.03	0.8	Female	West	Rural	Other CD	66	0.04	0.6	Female	West	Rural	EIB	39
Female	West	Rural	EIB	39	0.02	0.5	Female	West	Rural	EIB	40	0.02	0.4	Female	West	Rural	Pneumonia	37
Female	West	Rural	Suicide	•	•	•	Female	West	Rural	Meningitis	27	0.01	0.3	Female	West	Rural	Meningitis	30

Note:
Estimates are dashed-out if number of death is less than 25.

Table S4. National and subnational annual rate of reduction (ARR) by age and sex

Table S4.1. National all cause and cause-specific ARR

Causes of death	Male			Female		
	5-9 year	10-14 year	15-19 year	5-9 year	10-14 year	15-19 year
All causes	5.1	3.7	4.7	3.7	2.2	4.2
Congenital malformation	3.8	3.0	4.1	3.1	1.3	4.3
CVD	2.7	0.2	0.2	0.1	-0.3	0.3
Drowning	9.1	7.2	4.7	8.0	5.2	4.0
EIB	-2.1	0.1	0.1	-0.9	-3.4	0.2
Meningitis	5.0	4.0	7.1	4.6	7.4	10.2
Neoplasms	1.1	1.6	4.0	1.1	1.6	3.6
Other CD	7.9	8.8	6.7	9.1	6.0	9.8
Other Injuries	5.0	3.6	6.8	3.7	2.3	4.5
Other NCD	0.6	-1.8	1.4	0.0	-0.7	2.8
Pneumonia	6.2	4.0	3.0	5.6	2.7	2.4
Road traffic injury	5.9	3.5	6.9	3.9	2.7	5.0
Suicide	NA	2.8	3.5	NA	1.7	5.3

Table S4.2. Regional-residency strata level all cause and cause-specific ARR

Age group	Region	Residency	All causes	Congenital malformation	CVD	Drowning	EIB	Meningitis	Neoplasms	Other CD	Other Injuries	Other NCD	Pneumonia	Road traffic injury	Suicide
5-9	Central	Rural	3.7	4.6	1.5	6.8	-0.8	4.8	1.1	3.2	2.6	-1.2	5.6	4.8	NA
5-9	Central	Urban	3.9	0.9	2.4	10.0	1.4	-13.1	3.0	6.8	3.7	0.0	-11.2	6.2	NA
5-9	East	Rural	4.0	3.0	-5.1	9.9	-9.7	-0.3	0.0	7.0	3.6	2.7	2.9	5.4	NA
5-9	East	Urban	4.0	6.0	-3.2	10.2	1.5	2.9	3.5	0.8	3.6	-3.2	-1.0	5.7	NA
5-9	West	Rural	4.4	2.3	3.9	7.9	0.6	4.8	-0.3	10.5	4.6	-0.9	7.5	3.5	NA
5-9	West	Urban	5.1	2.6	4.3	7.8	-7.3	9.1	2.6	8.9	6.7	0.6	1.9	6.5	NA
10-14	Central	Rural	0.7	0.2	-4.9	3.4	-3.6	0.0	0.0	4.5	-0.3	-5.0	0.3	2.0	1.0
10-14	Central	Urban	3.1	1.7	-10.1	9.4	2.3	2.3	2.6	4.5	1.4	-2.3	-0.1	3.8	0.9
10-14	East	Rural	2.0	3.8	-2.0	6.9	-5.4	6.5	-0.7	4.9	1.2	-2.3	6.4	1.2	0.2
10-14	East	Urban	4.1	3.8	-2.4	11.5	5.4	-1.8	2.5	-4.7	4.1	0.4	-2.7	7.5	0.1
10-14	West	Rural	4.3	1.5	3.5	6.9	-1.3	8.2	3.3	10.3	5.4	0.0	5.2	3.1	4.4
10-14	West	Urban	5.0	3.7	8.2	5.5	-1.2	7.0	4.1	10.3	5.9	2.7	2.6	5.1	7.2
15-19	Central	Rural	3.8	2.6	0.1	2.1	0.1	12.2	3.3	8.4	5.3	1.1	2.1	6.1	4.1
15-19	Central	Urban	3.4	3.7	-2.8	6.0	-1.5	2.9	3.8	3.3	4.5	2.2	-4.2	4.6	3.5
15-19	East	Rural	6.0	6.4	0.0	6.2	-0.3	9.1	4.6	8.5	7.1	3.0	6.3	8.8	6.7
15-19	East	Urban	3.8	1.9	-0.5	3.3	0.7	-8.3	2.7	2.0	6.2	2.6	2.4	7.9	0.0
15-19	West	Rural	3.7	4.4	0.8	4.5	1.1	6.5	3.2	8.4	5.4	0.7	1.9	3.9	3.8
15-19	West	Urban	3.0	2.9	-1.4	2.6	-8.8	10.5	1.9	7.9	6.4	1.7	-2.7	3.8	1.2

Note:
CVD: Cardiovascular disease; Other CD: Other communicable, maternal, perinatal and nutritional diseases; Other NCD: Other non-communicable diseases.
